# Ti_3_C_2_ MXene co-catalyst on metal sulfide photo-absorbers for enhanced visible-light photocatalytic hydrogen production

**DOI:** 10.1038/ncomms13907

**Published:** 2017-01-03

**Authors:** Jingrun Ran, Guoping Gao, Fa-Tang Li, Tian-Yi Ma, Aijun Du, Shi-Zhang Qiao

**Affiliations:** 1 School of Chemical Engineering, The University of Adelaide, Adelaide, South Australia 5005, Australia; 2 School of Chemistry, Physics and Mechanical Engineering Faculty, Queensland University of Technology, Garden Point Campus, Brisbane, Queensland 4001, Australia; 3 College of Science, Hebei University of Science and Technology, Shijiazhuang 050018, China

## Abstract

Scalable and sustainable solar hydrogen production through photocatalytic water splitting requires highly active and stable earth-abundant co-catalysts to replace expensive and rare platinum. Here we employ density functional theory calculations to direct atomic-level exploration, design and fabrication of a MXene material, Ti_3_C_2_ nanoparticles, as a highly efficient co-catalyst. Ti_3_C_2_ nanoparticles are rationally integrated with cadmium sulfide via a hydrothermal strategy to induce a super high visible-light photocatalytic hydrogen production activity of 14,342 μmol h^−1 ^g^−1^ and an apparent quantum efficiency of 40.1% at 420 nm. This high performance arises from the favourable Fermi level position, electrical conductivity and hydrogen evolution capacity of Ti_3_C_2_ nanoparticles. Furthermore, Ti_3_C_2_ nanoparticles also serve as an efficient co-catalyst on ZnS or Zn_*x*_Cd_1−*x*_S. This work demonstrates the potential of earth-abundant MXene family materials to construct numerous high performance and low-cost photocatalysts/photoelectrodes.

## Abstract (web summary)

Solar hydrogen production through photocatalytic water splitting requires active and stable co-catalysts to replace platinum. Here, the authors use DFT to identify Ti_3_C_2_ nanoparticles as potential co-catalysts, and assess their photocatalytic hydrogen production activity.

The generation of hydrogen (H_2_) from water using solar energy is regarded as a promising strategy for solving global energy problems^1^,^2^,^3^. Particularly, photocatalytic water splitting by utilizing semiconductor photocatalysts has demonstrated huge potential as a clean, low-cost and sustainable approach for solar H_2_ production. However, despite tremendous achievement in this area during the past decades^1^,^4^,^5^, it is still a great challenge to develop highly efficient, cost-effective and robust photocatalysts driven by sunlight. In recent years, co-catalysts have shown great success in boosting both the activity and stability of photocatalysts^6^,^7^,^8^,^9^. Unfortunately, the high price and extreme scarcity of the most active H_2_ evolution co-catalyst, Pt, restricts the commercialization of current photocatalysts. Therefore, seeking an inexpensive and highly active co-catalyst to replace Pt is of paramount significance for achieving large-scale solar H_2_ production in the future.

To date, although enormous progress has been made in developing earth-abundant co-catalysts, several major problems, arising from the intrinsic properties of current co-catalysts, still exist: (i) lack of abundant surface functionalities to establish strong connection with photocatalysts, for fast interfacial charge transfer and long-term stability; (ii) inefficient electron shuttling within co-catalysts due to their poor semiconducting/insulating conductivity^10^ or destruction of π-conjugated system (for example, graphene oxide)^11^; (iii) undesirable Gibbs free energy for H_2_ evolution; (iv) insufficient contact with water molecules due to lack of hydrophilic functionalities; and (v) instability and/or requirement of non-aqueous environment (for example, hydrogenases and their mimics)^12^,^13^. Therefore, it is highly desirable to seek a brand-new family of materials as the next generation co-catalysts that can overcome these drawbacks.

MXene, a new family of over 60 two-dimensional (2D) metal carbides, nitrides or carbonitrides^14^,^15^, has shown great potential as electrodes in (Li)-ion batteries^16^ and supercapacitors^17^. Notably, their distinguished characteristics render them highly promising for solving the above problems as: (i) MXene possesses numerous hydrophilic functionalities (–OH and –O) on its surface, enabling it to easily construct strong connection with various semiconductors; (ii) the excellent metallic conductivity of MXene assures efficient charge-carrier transfer; (iii) the exposed terminal metal sites (for example, Ti, Nb or V) on MXene might lead to much stronger redox reactivity than that of the carbon materials^18^; (iv) the presence of numerous hydrophilic functionalities on MXene promotes its strong interaction with water molecules; and (v) MXene can stably function in aqueous solutions. Considering the above outstanding properties of the MXene family, it is anticipated that MXene will be a promising material to be employed in photocatalysis. However, to the best of our knowledge, there is no report on exploring MXene as a co-catalyst for photocatalysis.

Herein, we utilize density functional theory (DFT) calculations to explore the potential of Ti_3_C_2_ MXene as a H_2_ evolution co-catalyst. On the basis of theoretical studies, we report a rational design and synthesis of Ti_3_C_2_ nanoparticles (NPs) and merge them with a chosen photocatalyst, CdS, to successfully achieve a super high visible-light photocatalytic H_2_-production activity. The origin of this high activity is studied by both experimental techniques and theoretical investigations. Moreover, the general function of Ti_3_C_2_ NPs as an active co-catalyst for other photocatalysts is also confirmed, illustrating the considerable potential of MXene family materials to replace rare and costly Pt in photocatalysis/photoelectrocatalysis.

## Results

### Theoretical exploration of Ti_3_C_2_ MXene as a co-catalyst

**Figure 1:**
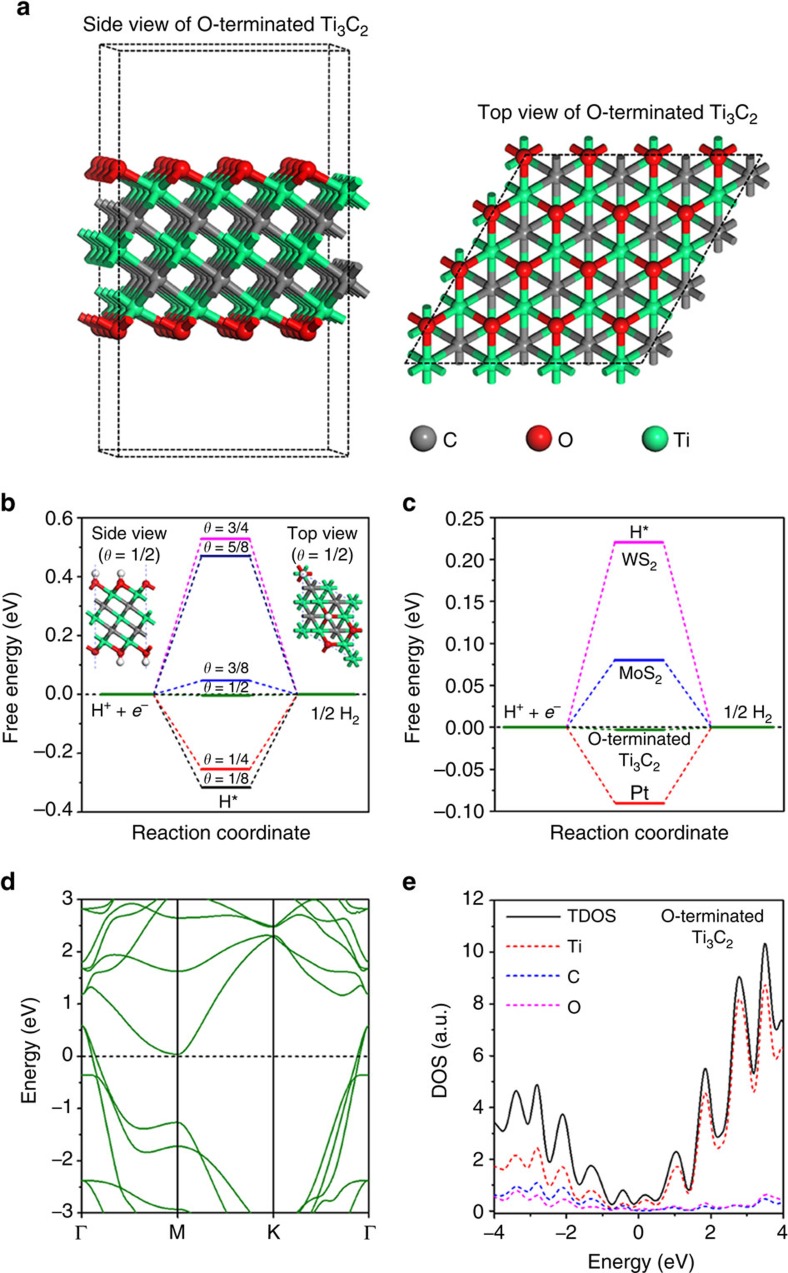
Density function theory calculation studies of O-terminated Ti_3_C_2_. (**a**) The side and top views of the structure model for a 4 × 4 × 1 O-terminated Ti_3_C_2_ supercell. Grey, red and cyan spheres denote C, O and Ti atoms, respectively. (**b**) The calculated free-energy diagram of HER at the equilibrium potential (*U*=0 V) on the surface of a 2 × 2 × 1 O-terminated Ti_3_C_2_ supercell at different H* coverage (1/8, 1/4, 3/8, 1/2, 5/8 and 3/4) conditions (the side and top views of a 2 × 2 × 1 O-terminated Ti_3_C_2_ supercell at 1/2 H* coverage are shown in the inset). (**c**) The calculated free-energy diagram of HER at the equilibrium potential (U=0 V) on the surface of a 2 × 2 × 1 O-terminated Ti_3_C_2_ supercell at 1/2 H* coverage, and the referenced Pt (ref. 21, 22) MoS_2_ (ref. 23), and WS_2_ (ref. 23). (**d**) The calculated band structure of O-terminated Ti_3_C_2_. (**e**) The total density of states (TDOS) and partial density of states (PDOS) for O-terminated Ti_3_C_2_.

To explore the possibility of using Ti_3_C_2_ MXene as a highly efficient and low-priced co-catalyst to promote H_2_ production, we have conducted a series of theoretical investigations based on DFT calculations. A highly active co-catalyst can not only rapidly extract photo-induced electrons from a photocatalyst to its surface, but also efficiently catalyse the H_2_ evolution on its surface, by using those electrons^6^. Herein, we first focus on the H_2_ evolution activity to evaluate whether Ti_3_C_2_ is an excellent candidate. Usually, the overall H_2_ evolution reaction (HER) pathway can be summarized by a three-state diagram, composed of an initial state H^+^+*e*^−^, an intermediate adsorbed H*, and a final product ½H_2_ (refs 19, 20). The Gibbs free energy of the intermediate state, |Δ*G*_H*_|, is regarded as a major indicator of the HER activity for various catalysts. The most desirable value for |Δ*G*_H*_| should be zero^20^. For example, the highly active and well-known HER catalyst, Pt, shows a near-zero value of Δ*G*_H*_≈−0.09 eV (refs 21, 22). Thus, we performed DFT studies to calculate Δ*G*_H*_ for atomic H adsorption on the surface of O-terminated Ti_3_C_2_, pure Ti_3_C_2_ and F-terminated Ti_3_C_2_, respectively. Their structural models are displayed in Fig. 1a and Supplementary Figs 1,2, respectively. Pure Ti_3_C_2_ exhibits a largely negative Δ*G*_H*_=−0.927 eV (Supplementary Fig. 3a), suggesting too strong chemical adsorption of H* on its surface. Meanwhile, a largely positive Δ*G*_H*_=1.995 eV is observed for F-terminated Ti_3_C_2_ (Supplementary Fig. 3b), indicating very weak H* adsorption and easy product desorption. Unfortunately, both conditions are unfavourable for HER. Surprisingly, O-terminated Ti_3_C_2_ shows a near-zero value of |Δ*G*_H*_|=0.00283, eV at its optimal H* coverage (*θ*=1/2) (Fig. 1b; Supplementary Table 1). This value is even much lower than that of Pt or highly active earth-abundant HER catalysts (Fig. 1c), for example, MoS_2_ (Δ*G*_H*_=0.08 eV)^23^ or WS_2_ (Δ*G*_H*_=0.22 eV)^23^, clearly indicating the remarkable HER activity of O-terminated Ti_3_C_2_ from the viewpoint of thermodynamics.

Apart from extraordinary HER activity, a highly active co-catalyst must efficiently extract the photo-induced electrons from photocatalysts and deliver them to its surface, which requires appropriate electronic band structure and excellent conductivity. Hence, we employ DFT calculations to determine the band structures of Ti_3_C_2_, F-terminated Ti_3_C_2_ and O-terminated Ti_3_C_2_, respectively. As shown in Supplementary Fig. 4a,b, pure Ti_3_C_2_ exhibits metallic characteristics with substantial electronic states crossing the Fermi level. In comparison, F-terminated Ti_3_C_2_ (Supplementary Fig. 4c,d) and O-terminated Ti_3_C_2_ (Fig. 1d,e) exhibit decreased numbers of states at the Fermi level, indicating their lower conductivities. Nevertheless, the continuous electronic states crossing Fermi level for F-terminated Ti_3_C_2_ and O-terminated Ti_3_C_2_ indicate that their conductivities are still good. Hence, Ti_3_C_2_ retains its outstanding electrical conductivity, even after decoration with numerous functionalities, implying its exceptional capability to transport electrons. We believe this unique merit of MXene renders it a superior co-catalyst outperforming its counterparts, such as graphene and carbon nanotubes, which suffer obvious conductivity loss after their termination with –O, –OH and –COO^−^ (ref. 11). Furthermore, the Fermi levels (*Ε*_F_) of Ti_3_C_2_, O-terminated Ti_3_C_2_ and F-terminated Ti_3_C_2_ are calculated to be −0.05 V, 1.88 V and 0.15 V versus SHE, respectively. Among them, O-terminated Ti_3_C_2_ displays the most positive value of *E*_F_, implying its strongest capacity to accept photo-induced electrons from semiconductor photocatalysts.

On the basis of the above theoretical explorations, it can be concluded that both pure Ti_3_C_2_ and F-terminated Ti_3_C_2_ are not eligible candidates due to their inefficient HER activity and unfavourable *E*_F_. In contrast, O-terminated Ti_3_C_2_ is predicted to be a highly promising co-catalyst, given its outstanding HER activity, excellent metallic conductivity and desirable *E*_F_.

### Design and synthesis of Ti_3_C_2_-incorporated CdS

The above theoretical investigations provide clear guidance to synthesize Ti_3_C_2_ co-catalyst and couple it with photocatalysts. Firstly, we need to obtain Ti_3_C_2_ terminated with abundant functionalities instead of pure Ti_3_C_2_. Then, we should minimize and maximize the number of –F and –O terminations on Ti_3_C_2_, respectively. To achieve this goal, as presented in Supplementary Fig. 5, Ti_3_AlC_2_ (MAX phase) powders were firstly etched by HF to remove Al species, producing exfoliated Ti_3_C_2_ (Ti_3_C_2_-E) with an accordion-like architecture (Supplementary Fig. 6a). During the etching process, Ti_3_C_2_-E was spontaneously decorated with substantial functionalities (–OH, –F and –O) on its surface, giving rise to its exceptional hydrophilicity. The transformation from Ti_3_AlC_2_ to Ti_3_C_2_ is firmly evidenced by the obvious shift of the (002) and (004) X-ray diffraction (XRD) peaks to lower degrees, and the disappearance of the strongest diffraction peak of Ti_3_AlC_2_ at 39° (Supplementary Fig. 7)^24^. To further increase the surface area and functionalities of Ti_3_C_2_, Ti_3_C_2_-E was added to de-ionized water and subjected to strong ultra-sonication, during which many large Ti_3_C_2_-E sheets were cut into small pieces of Ti_3_C_2_ NPs. The resulting suspension was centrifuged at 10,000 r.p.m. to remove the large Ti_3_C_2_ sheets and particles, leaving the small Ti_3_C_2_ NPs in the supernatant (Supplementary Fig. 8a). The successful formation of Ti_3_C_2_ NPs is supported by the XRD pattern (Supplementary Fig. 7; Supplementary Note 1), high-angle annular dark-field (HAADF) image (Supplementary Fig. 8b), energy-dispersive X-ray spectra (EDX) elemental mapping images (Supplementary Fig. 8c–f), X-ray photoelectron spectroscopy (XPS) survey spectrum (Supplementary Fig. 9a), and high-resolution XPS spectra of Ti 2*p*, O 1*s* and F 1*s* (Supplementary Fig. 9b,c,d). The presence of abundant hydrophilic functionalities (–O, –OH and –F) on Ti_3_C_2_ NPs is supported by the high-resolution XPS spectrum of O 1*s* and F 1*s* (Supplementary Fig. 9c,d). Meanwhile, the ‘black’ colloid dispersion of Ti_3_C_2_ exhibits a typical Tyndall effect (Supplementary Fig. 8a, inset), reasonably suggesting the formation of a homogeneous dispersion of Ti_3_C_2_ NPs. The engineering of three-dimensional (3D) Ti_3_C_2_-E into zero-dimensional (0D) Ti_3_C_2_ NPs dramatically increased their surface area and functionalities, thus greatly favoring their intimate coupling with photocatalysts.

Then, CdS was selected as the photocatalyst to couple with Ti_3_C_2_, since its reported conduction band (CB) potential (−0.7 V versus SHE)^25^ is much more negative than the *E*_F_ of O-terminated Ti_3_C_2_ (1.88 V versus SHE). Besides, to obtain the desired functionalities on Ti_3_C_2_, a hydrothermal strategy is applied to integrate CdS with Ti_3_C_2_ NPs. So the –F terminations can be replaced by –O or –OH in the aqueous environment during hydrothermal treatment. The synthesis process is shown in Supplementary Fig. 10. Ti_3_C_2_ NPs were firstly introduced into Cd(Ac)_2_ aqueous solution, in which Cd^2+^ cations were easily adsorbed on numerous -O terminations. Then, an organic sulfur source, thiourea, was added into the above suspension and coordinated with Cd^2+^. Finally, the resulting suspension was subjected to hydrothermal treatment. During this process, most of the –F terminations on Ti_3_C_2_ NPs were replaced by –O/–OH terminations, and thiourea molecules decomposed to gradually release S^2−^ anions into the solution. These S^2−^ anions were combined with the Cd^2+^ cations adsorbed on the surface of Ti_3_C_2_ NPs, leading to the heterogeneous nucleation and growth of CdS NPs on Ti_3_C_2_ NPs. Meanwhile, the excessive Cd^2+^ cations were also combined with these S^2−^ anions, resulting in the homogeneous nucleation and growth of pure CdS NPs. Then both CdS/Ti_3_C_2_ nanocomposites and CdS NPs self-assembled to form a large cauliflower-structured CdS/Ti_3_C_2_ sub-microsphere (SMS), with Ti_3_C_2_ NPs intimately coupled. The nominal mass ratios of Ti_3_C_2_ to CdS were 0, 0.05, 0.1, 2.5, 5 and 7.5 wt.%, and the resulting samples were labelled as CT0, CT0.05, CT0.1, CT2.5, CT5 and CT7.5, respectively. The actual mass ratios of the synthesized samples were determined by inductively coupled plasma atomic emission spectrometry (ICP-AES) (Supplementary Table 2).

### Chemical composition and morphology

The chemical composition and morphology of the as-prepared samples were thoroughly investigated. Firstly, their crystal structures were characterized by XRD. The XRD patterns (Supplementary Fig. 11a) confirm that all the samples are composed of hexagonal wurtzite-structured phase CdS (JCPDS No. 77-2306). A combination of Ti_3_C_2_ NPs with CdS did not affect the crystal structure of CdS, suggesting that the remarkable increase in photocatalytic activity is not caused by any crystal structure alteration in CdS. Instead, it should be attributed to the Ti_3_C_2_ NPs deposited on its surface. However, no diffraction peaks for Ti_3_C_2_ are observed in Supplementary Fig. 11a, probably due to the low loading and high dispersion of Ti_3_C_2_ NPs on the surface of CdS.

**Figure 2:**
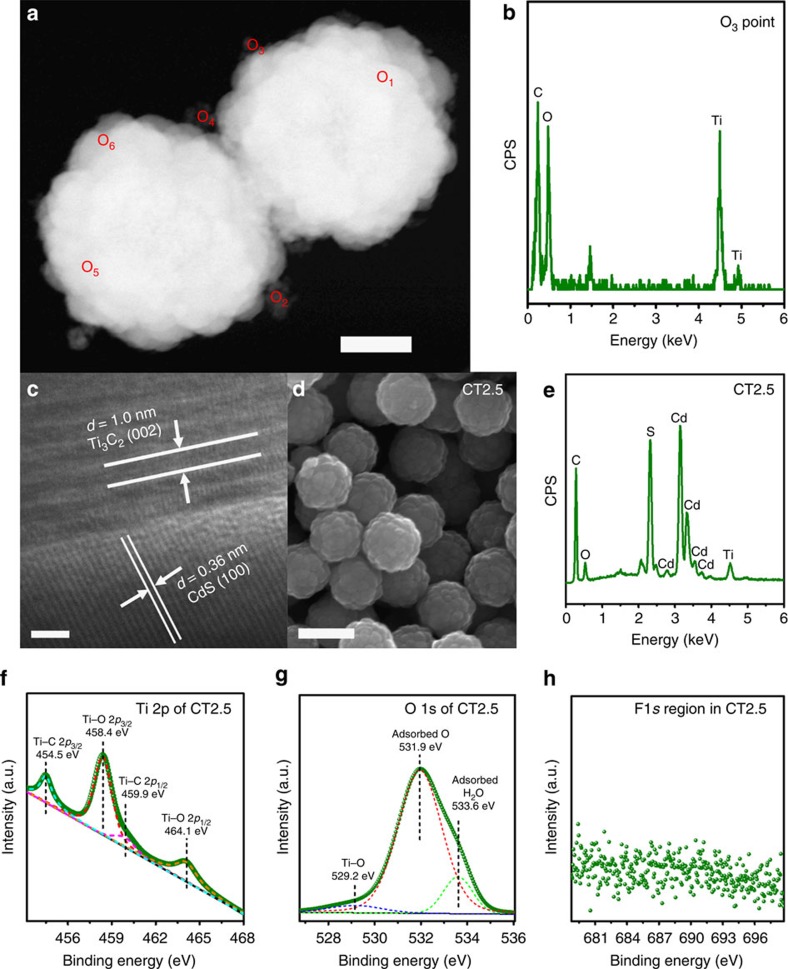
Morphology and chemical composition of CT2.5. (**a**) A typical high-angle annular dark-field (HAADF) image of CT2.5 and the six different points (O_1_, O_2_, O_3_, O_4_, O_5_ and O_6_) for EDX analysis. (**b**) The EDX spectrum at O_3_ point in **a**. (**c**) The high-resolution TEM image near O_3_ point in **a**. (**d**,**e**) A typical SEM image of CT2.5 and its corresponding EDX spectrum. (**f**–**h**) The high-resolution XPS spectra of Ti 2*p*, O 1*s* and F 1*s* for CT2.5. Scale bars, 200 nm (**a**), 2 nm (**c**) and 500 nm (**d**).

The morphology and composition of the as-synthesized CT2.5 were further investigated by HAADF, EDX, high-resolution (HR)TEM, SEM and XPS techniques. The HAADF image of CT2.5 in Fig. 2a show that several NPs are deposited on the surface of CdS SMS, which is quite different from the smooth surface of pure CdS SMS (CT0) displayed in Supplementary Fig. 12a,b. The composition of these NPs was *in situ* studied by EDX and HRTEM. Firstly, three points of O_2_, O_3_ and O_4_ at these NPs were selected for EDX analysis, respectively. The results in Fig. 2b and Supplementary Fig. 13b,c exhibit that Ti peaks were found, while no Cd or S peaks were observed at O_2_, O_3_ and O_4_, suggesting that these NPs are not CdS but Ti-containing material. The HRTEM image near the O_3_ point (Fig. 2c) shows a hetero-interface with lattice spacings of 1 and 0.36 nm, which are assigned to the (002) plane of Ti_3_C_2_ (ref. 24) and (100) plane of CdS^26^, respectively. This result confirms the formation of CdS/Ti_3_C_2_ hetero-junction. Furthermore, the SEM image of CT2.5 in Fig. 2d shows a uniform SMS structure of CdS/Ti_3_C_2_ with sizes of ca. 400–500 nm. A detailed observation in Fig. 2d suggests that CdS/Ti_3_C_2_ SMS has a cauliflower-structured morphology created by the self-assembly of many NPs^27^. The corresponding EDX spectrum in Fig. 2e indicates that CT2.5 contains Cd, S, Ti and C, which is consistent with the HRTEM image and EDX spectra. The above results support the establishment of intimate coupling between Ti_3_C_2_ and CdS, implying the efficient interfacial photo-induced charge diffusion on visible-light irradiation^7^,^28^. Moreover, the high-resolution XPS spectrum of Ti 2*p* exhibits four deconvoluted peaks in Fig. 2f, corresponding to Ti–O 2*p* and Ti–C 2*p*^24^, in agreement with the above HRTEM and EDX results. It should be noted that numerous –O terminations are present in CT2.5 (Fig. 2g), while the F content is negligible for CT2.5 (Fig. 2h), suggesting the successful replacement of –F by –O/–OH on Ti_3_C_2_ NPs after hydrothermal treatment. Thus, the ratio of F to O in CT2.5 is zero.

### Super high photocatalytic H_2_-production performance

**Figure 3:**
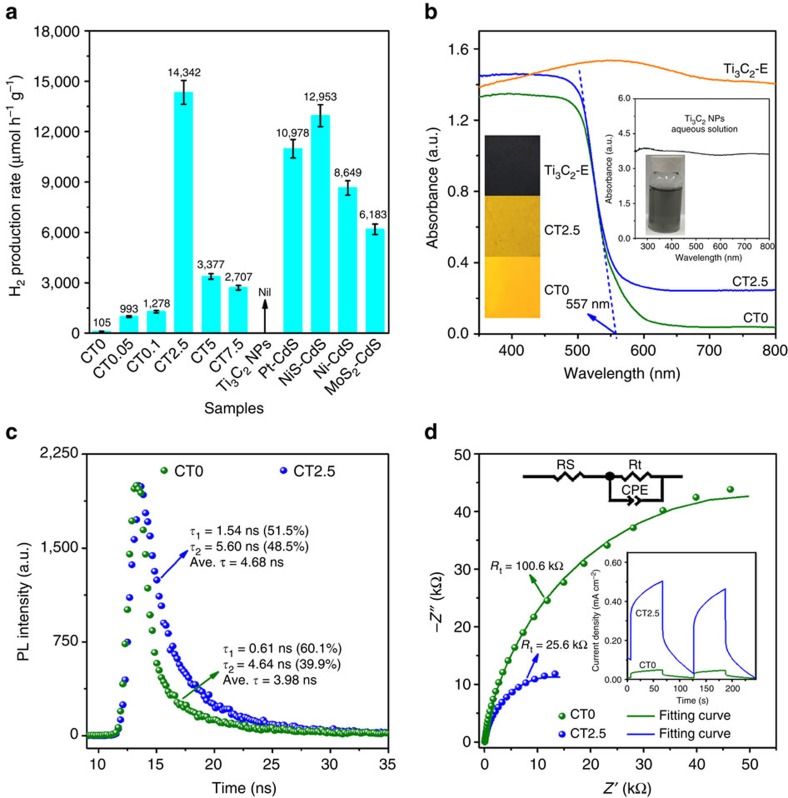
Photocatalytic performance and spectroscopy/(photo)electrochemical characterization. (**a**) A comparison of the photocatalytic H_2_-production activities of CT0, CT0.05, CT0.1, CT2.5, CT5, CT7.5, Ti_3_C_2_ NPs, Pt–CdS, NiS–CdS, Ni–CdS and MoS_2_–CdS. The error bars are defined as s.d. (**b**) Ultraviolet-visible diffuse reflectance spectra of CT0, CT2.5 and Ti_3_C_2_-E. The insets show the colours of all the samples as well as the ultraviolet-visible absorbance spectrum and picture of the Ti_3_C_2_ NPs aqueous solution. (**c**) Time-resolved PL spectra of CT0 and CT2.5. (**d**) EIS Nyquist plots of CT0 and CT2.5 electrodes measured under the open-circle potential and visible-light irradiation in 0.5 M potassium phosphate buffer (pH=7) solution. The inset shows the transient photocurrent responses of CT0 and CT2.5 electrodes in 0.2 M Na_2_S+0.04 M Na_2_SO_3_ mixed aqueous solution under visible-light irradiation.

The photocatalytic H_2_-production activity of all the as-prepared samples was examined in 18 vol.% lactic acid aqueous solution under visible-light irradiation (*λ*≥420 nm). Excitingly, the coupling of Ti_3_C_2_ NPs with CdS indeed leads to a remarkable enhancement in the photocatalytic activity. As displayed in Fig. 3a, pristine CdS (CT0) shows a very low photocatalytic activity of 105 μmol h^−1 ^g^−1^. In contrast, the loading of a small amount of Ti_3_C_2_ NPs (0.05 wt.%) obviously improves the photocatalytic activity of CT0.05 to 993 μmol h^−1 ^g^−1^. With increasing amount of Ti_3_C_2_ NPs, the photocatalytic activity of Ti_3_C_2_-loaded CdS is gradually enhanced. Surprisingly, a super high photocatalytic H_2_-production activity of 14,342 μmol h^−1^ g^−1^ is achieved on CT2.5, exceeding that of CT0 by an amazing factor of 136.6. In comparison, for the same loading (2.5 wt.%) and experimental conditions, NiS, Ni and MoS_2_-loaded CdS SMS (NiS–CdS, Ni–CdS and MoS_2_–CdS) exhibit lower photocatalytic activities of 12,953, 8,649 and 6,183 μmol h^−1 ^g^−1^, respectively (Fig. 3a). Besides, CT2.5 also shows higher quantum efficiency (40.1% at 420 nm) than the other noble-metal-free CdS-based photocatalysts reported to date, such as: Ni/CdS, Ni(OH)_2_/CdS, Ni_2_P/CdS, CoP/CdS, graphene oxide/CdS and MoS_2_/CdS (Supplementary Table 3). On the basis of the above experimental data and literature, Ti_3_C_2_ NPs have proven to be one of the most active earth-abundant co-catalysts. Furthermore, CT2.5 even displays higher activity than 2.5 wt.% Pt loaded CdS SMS (Pt–CdS, 10,978 μmol h^−1 ^g^−1^), even though Pt is widely accepted as the most active co-catalyst promoting H_2_ production. The HAADF image, EDX elemental mapping images, TEM and HRTEM images of Pt–CdS (Supplementary Fig. 14a–f) imply that Pt is homogeneously decorated on CdS in the form of clusters (Supplementary Note 2). The size of Pt in Pt–CdS is much smaller than that of Ti_3_C_2_ in CT2.5, suggesting more active sites exposed on Pt than those on Ti_3_C_2_ for the same loading. In this case, the superior activity of CT2.5 should be ascribed to the much stronger combination between CdS and Ti_3_C_2_ established during hydrothermal treatment, which greatly facilitates the rapid interfacial charge transfer^7^,^28^. This result also highlights the huge potential of Ti_3_C_2_ NPs as a high performance and low-cost co-catalyst to replace Pt. However, further increase in the loading of Ti_3_C_2_ NPs leads to the drastic deterioration of photocatalytic activity as reported in previous works^6^,^7^,^28^,^29^. This is due to the excessive Ti_3_C_2_ NPs covering the surface active sites and impeding the light absorption of CdS. Nevertheless, CT7.5 still retains a photocatalytic activity of 2,707 μmol h^−1 ^g^−1^, much higher than that of CT0. In addition, Ti_3_C_2_ NPs show no activity toward H_2_ production under visible-light irradiation, further supporting its role as a co-catalyst rather than a photocatalyst.

The stability of the optimized CT2.5 was further evaluated by performing the photocatalytic experiments under the same reaction conditions for seven cycles. No significant deterioration of photocatalytic activity was observed for CT2.5 during seven successive cycling tests for H_2_ production (Supplementary Fig. 15a). A comparison of the crystalline phase (Supplementary Fig. 11a), morphology and size (Fig. 2a and Supplementary Fig. 15b) between the original and used CT2.5 (CT2.5-A) shows no apparent alterations in CT2.5-A, which is in accordance to its repeated high activity.

### Light-harvesting capability

To investigate the origin of the remarkable activity of CT2.5, its properties governing the three major processes in photocatalytic reactions (that is, light absorption, charge separation and transfer, and surface redox reactions^1^,^4^,^5^,^6^) were thoroughly characterized. Firstly, the light-harvesting capability of CT2.5 was measured by the ultraviolet-visible diffuse reflectance spectra. As displayed in Fig. 3b, the light absorption of CT2.5 is obviously increased throughout the entire region of 350–800 nm, due to the black colour of loaded Ti_3_C_2_ NPs (Fig. 3b, inset). Similar phenomenon is also observed for CT0.05, CT0.1, CT5 and CT7.5 (Supplementary Fig. 11b). The ultraviolet-visible absorbance spectrum of the Ti_3_C_2_ NPs aqueous solution shows no obvious absorption edge in the 250–800 nm region, implying the metallic nature of Ti_3_C_2_ NPs. Furthermore, no apparent shift in the absorption edge of CT2.5 is observed, indicating that Ti, C, F or O element is not doped into the crystal structure of CdS, which is in agreement with the above XRD data. To investigate whether the increased visible-light absorption originating from Ti_3_C_2_ NPs enhanced the photocatalytic activity of CT2.5, a 560 nm light filter was employed to cutoff any irradiation light with wavelength shorter than 557 nm (the onset absorption edge of CdS in CT2.5), while other experimental conditions were kept identical. Under such conditions, CT2.5 shows no activity for H_2_ production, indicating that the enhanced visible-light absorption arising from Ti_3_C_2_ NPs is unlikely to promote the activity enhancement observed for CT2.5.

### Charge separation and transfer

To study the charge-carrier separation and transfer efficiency in CT2.5, a series of characterization techniques including time-resolved and steady-state photoluminescence (PL) spectra, electrochemical impedance spectra (EIS) and transient photocurrent (TPC) response were used. As shown in Fig. 3c, in comparison to CT0, CT2.5 shows an increased short (*τ*_1_), long (*τ*_2_) and intensity-average (*τ*) PL lifetimes, indicating that the deposition of Ti_3_C_2_ on CdS can effectively suppress the charge recombination and elongate the lifetime of charge carriers. The enhanced charge separation efficiency is further confirmed by the quenched emission peak around 560 nm for CT2.5 (Supplementary Fig. 16). Furthermore, the surface and bulk charge-transfer efficiencies were investigated by the EIS and TPC density measurements, respectively. As indicated in Fig. 3d, CT2.5 shows a much smaller semicircle diameter and a much lower interfacial charge-transfer resistance than those of CT0 in potassium phosphate buffer solution (pH=7) under visible-light irradiation, suggesting the apparent enhancement of interfacial charge-carrier transfer on the surface of CdS/Ti_3_C_2_. On the other hand, to study the bulk charge transfer in CT0 and CT2.5, the TPC density measurements were conducted. Na_2_S and Na_2_SO_3_ were applied as electrolytes to rapidly capture the photo-induced holes on the surface of CT0 and CT2.5. Thus, these hole scavengers were supposed to eliminate the surface charge recombination on CT0 and CT2.5. In such a case, the observed enhancement in the TPC density on loading of Ti_3_C_2_ (Fig. 3d, inset) directly reflects an improved charge separation efficiency in the bulk of CdS/Ti_3_C_2_.

To gain further insights into the charge separation and transfer mechanism in CT2.5, the CB and valence band (VB) potentials of CdS in CT2.5 were determined to be −0.79 V and 1.54 V versus SHE, respectively, by a combination of Mott-Schottky and Tauc plots (Supplementary Fig. 17a,b). Hence, on light irradiation, the photo-induced electrons on the CB of CdS (*E*_CB_=−0.79 V versus SHE) in CT2.5 can promptly migrate to O-terminated Ti_3_C_2_ NPs, which rapidly shuttle these photo-induced electrons to their surface active sites, because of their low *E*_F_ position and excellent conductivity. Therefore, in the case of CT2.5, Ti_3_C_2_ can serve as an electron trapping and shuttling site not only to suppress the charge recombination on the surface of CdS, but also to promote the charge separation and transfer in the bulk of CdS, which is consistent with the above results.

### Surface catalytic redox reactions

Following the charge separation and transfer, the last step in photocatalytic H_2_ production includes the surface redox reactions catalysed by the reactive sites on CT2.5. Therefore, to study the efficiency of the last step, we determined the specific surface area and pore volume of all the samples by N_2_ sorption analysis (Supplementary Fig. 18a,b). As shown in Supplementary Table 2, an initial increase in the loading of Ti_3_C_2_ NPs up to 1.89 wt.% (CT0.05, CT0.1 and CT2.5) caused a gradual enlargement in the specific surface area of the CdS/Ti_3_C_2_ composites. However, further increase in the loading of Ti_3_C_2_ NPs resulted in a noticeable decrease in surface area to 3.8 and 3.7 m^2^ g^−1^ for CT5 and CT7.5, respectively, despite that Ti_3_C_2_ NPs exhibit a large surface area of 120.1 m^2 ^g^−1^ (Supplementary Table 4). This decrease is observed at higher loadings of Ti_3_C_2_ NPs because of their tendency to aggregate on the surface of CdS SMS. Hence, the highest surface area of CT2.5 among all the CdS/Ti_3_C_2_ composites suggests the existence of abundant active sites on its surface, which greatly promote the surface redox catalytic reactions. Moreover, the polarization curves of CT0, CT2.5 and Ti_3_C_2_ NPs (Supplementary Fig. 19) indicate that the presence of Ti_3_C_2_ NPs on the surface of CdS can greatly improve the HER activity of CT2.5, and consequently, contribute to its enhanced photocatalytic H_2_ production.

To further reveal the differences in HER mechanistic behaviour between Ti_3_C_2_ and other state-of-the-art earth-abundant HER catalysts, for example, MoS_2_ and WS_2_, DFT calculations were conducted to study the effect of H_2_ coverage on Δ*G*_H*_ for O-terminated Ti_3_C_2_. Fig. 1b shows that one O-terminated Ti_3_C_2_ unit cell tends to allow for adsorption of four H* due to its smallest |Δ*G*_H*_| (Supplementary Note 3), corresponding to the unsaturated H* coverage of *θ*=1/2. The |Δ*G*_H*_| values for the adsorption of H* on O-terminated Ti_3_C_2_ at *θ* values below 1/2 (that is, *θ*=1/8, 1/4 and 3/8) are relatively low (Supplementary Table 1). However, the further increase of H* coverage results in a rapid increase of |Δ*G*_H*_| and deterioration of HER activity (Fig. 1b; Supplementary Table 1). Nevertheless, O-terminated Ti_3_C_2_ still possesses a relatively large number of HER active sites considering its large surface with numerous active sites. In comparison, the HER active sites of well-known MoS_2_ and WS_2_ are only located at the edge positions, while all the sites in the basal plane are inactive^30^, suggesting the superiority of this newly developed O-terminated Ti_3_C_2_.

### Photocatalytic H_2_-production mechanism and discussion

**Figure 4:**
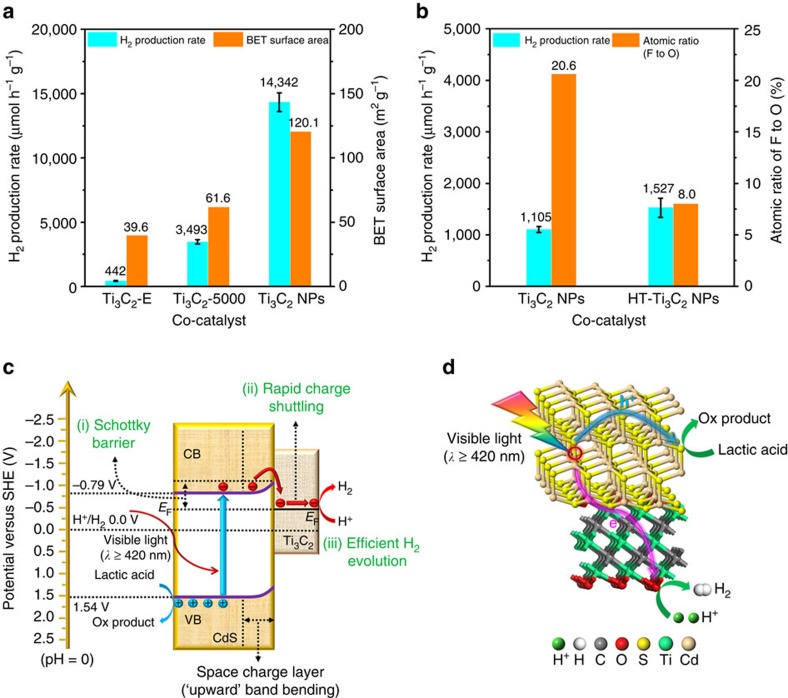
Origin and mechanism of the enhanced photocatalytic performance in CdS/Ti_3_C_2_ system. (**a**) The influence of the co-catalyst’s surface area on the photocatalytic activity. The error bars are defined as s.d. (**b**) The influence of the co-catalyst’s surface F to O atomic ratio on the photocatalytic activity. The error bars are defined as s.d. (**c**) The charge separation and transfer in the CdS/Ti_3_C_2_ system under visible-light irradiation. Red and blue spheres denote photo-induced electrons and holes, respectively. (**d**) Proposed mechanism for photocatalytic H_2_ production in the CdS/Ti_3_C_2_ system under visible-light illumination. Green sphere denotes H^+^. White, grey, red, yellow, cyan and gold spheres denote H, C, O, S, Ti and Cd atoms, respectively.

To gain an insight into the influence of intrinsic properties of Ti_3_C_2_ on the photocatalytic activity of the CdS/Ti_3_C_2_ composite, a series of experiments were designed and conducted. Firstly, the effect of co-catalyst’s surface area on the activity was studied. Co-catalysts Ti_3_C_2_-E, Ti_3_C_2_-5000 and Ti_3_C_2_ NPs with different sizes (Supplementary Figs 6a, 8a and 20) and corresponding surface areas (Supplementary Table 4) were respectively coupled with CdS at the same loading (2.5 wt.%) under identical hydrothermal conditions. As shown in Fig. 4a, loading Ti_3_C_2_-E, Ti_3_C_2_-5000 and Ti_3_C_2_ NPs with increasing surface area leads to gradually enhanced photocatalytic activities. This is because the smaller size and larger number of exposed active sites of Ti_3_C_2_ not only result in stronger coupling with CdS, but also assure better access to reactants. Secondly, the influence of functionalities of co-catalyst on the activity of CdS/Ti_3_C_2_ was investigated. Ti_3_C_2_ NPs were subjected to a hydrothermal treatment to reduce the number of –F terminations. The surface atomic ratio of F to O, estimated by XPS analysis, for Ti_3_C_2_ NPs and hydrothermally treated Ti_3_C_2_ NPs (HT-Ti_3_C_2_ NPs) are 20.6% and 8.0%, respectively. This implies that a large number of the -F terminations were exchanged into –O/–OH terminations for HT-Ti_3_C_2_ NPs during hydrothermal treatment. Then Ti_3_C_2_ NPs and HT-Ti_3_C_2_ NPs were mechanically mixed with CT0 at the same loading (2.5 wt.%), respectively. Figure 4b displays that HT-Ti_3_C_2_ NPs induce a higher photocatalytic activity of 1,527 μmol h^−1 ^g^−1^ than Ti_3_C_2_ NPs (1,105 μmol h^−1 ^g^−1^), even though the surface area of HT-Ti_3_C_2_ NPs (56.7 m^2 ^g^−1^) is much lower than that of Ti_3_C_2_ NPs (120.1 m^2 ^g^−1^) as shown in Supplementary Table 4. The reason for this is that the replacement of –F by –O/–OH on Ti_3_C_2_ NPs increases the density of effective active sites (–O terminations), despite the decreased surface area after hydrothermal treatment. This result coincides with the above DFT calculation data of Δ*G*_H*_ on O-terminated and F-terminated Ti_3_C_2_.

On the basis of the above experimental results and theoretical calculations, a photocatalytic mechanism illustrating the surprisingly high photocatalytic H_2_-production activity of CT2.5 is proposed in Fig. 4c,d. Since the original *E*_F_ of *n*-type CdS (slightly lower than its CB position of −0.91 V versus SHE) is much more negative than the original *E*_F_ of O-terminated Ti_3_C_2_ (1.88 V versus SHE), the intimate contact between CdS and Ti_3_C_2_ in CT2.5 leads to the electron transfer from CdS to Ti_3_C_2_ (Supplementary Note 4), accompanied by the rise of *E*_F_ for Ti_3_C_2_ above the hydrogen evolution potential (0.00 V versus SHE) and the equilibrium of *E*_F_ in CdS/Ti_3_C_2_ system. The similar phenomenon was reported by Jakob *et al*.^31^. Moreover, the CB position of CdS in CT2.5 is also lowered to −0.79 V versus SHE as confirmed in Supplementary Fig. 17a. Meanwhile, the immobilized positive charges remain in CdS near the CdS/Ti_3_C_2_ interface, where a space charge layer is formed, and the CB and VB of CdS are bent ‘upward’. Hence, a Schottky junction is formed between Ti_3_C_2_ and CdS. On visible-light (*λ*≥420 nm) irradiation, the electrons are excited from the VB to the CB of CdS. Due to the reduced space charge layer thickness in nano-sized CdS primary particles, the ‘upward’ bending of the CB and VB for CdS is also limited (Fig. 4c)^32^. Hence, the photo-induced electrons in the CB can still migrate across the ‘upward’ bent CB to the Fermi level of Ti_3_C_2_, leaving the photo-induced holes in the VB of CdS. As a result, the Schottky junction can serve as an electron trap to efficiently capture the photo-induced electrons, without impeding the electron transfer from CdS to Ti_3_C_2_, as reported in previous works^33^,^34^,^35^. After being transferred to Ti_3_C_2_, the photo-induced electrons are further rapidly shuttled to its surface, due to the excellent metallic conductivity. Finally, thanks to the outstanding HER capacity of Ti_3_C_2_, the protons in the aqueous solution are efficiently reduced by the photo-induced electrons at the abundant –O terminations on Ti_3_C_2_ to evolve H_2_ gas. Therefore, through tuning the number and type of surface functionalities on Ti_3_C_2_, one can achieve the desirable *E*_F_ and optimize the HER activity for Ti_3_C_2_, which imposes a pronounced synergetic enhancement effect on the photocatalytic activity of the CdS/Ti_3_C_2_ system.

The potential of this newly developed co-catalyst can be further exploited by a co-loading strategy. For instance, a *p*-type semiconductor NiS could be simultaneously loaded with Ti_3_C_2_ NPs on CdS SMS. Surprisingly, the photocatalytic activity of CdS/1 mol.% NiS/2.5 wt.% Ti_3_C_2_ (CNT2.5) was further increased to 18,560 μmol h^−1 ^g^−1^ as presented in Supplementary Fig. 21a. This is because the combination of *p*-type NiS with *n*-type CdS results in the formation of a *p–n* junction, which promotes the transfer of photo-induced holes from CdS to NiS. Meanwhile, the photo-induced electrons are rapidly extracted from CdS to Ti_3_C_2_ NPs for H_2_ evolution. Therefore, the co-loading strategy imposes a strong synergistic effect on the charge separation and transfer in CNT2.5, which is confirmed by combined techniques of PL spectra (Supplementary Fig. 22a) and TPC response (Supplementary Fig. 22b). These results demonstrate the great potential of co-loading Ti_3_C_2_ with other co-catalysts to achieve synergetic enhancement of photocatalytic activity.

### Ti_3_C_2_ as a versatile HER co-catalyst

To verify that the Ti_3_C_2_ NPs can act as a versatile HER co-catalyst on different photocatalysts, we mechanically mixed Ti_3_C_2_ NPs with Zn_*x*_Cd_1−*x*_S and ZnS respectively, and tested the photocatalytic H_2_-production activity of the resultant mixtures. As shown in Supplementary Figs 23a and 24a, a simple mechanical mixing of Zn_0.8_Cd_0.2_S (ZCS) and ZnS with 1 wt.% Ti_3_C_2_ NPs increased the photocatalytic activities of the formed composites ZCS/Ti_3_C_2_ and ZnS/Ti_3_C_2_ by 386 and 217%, respectively, as compared with that of pristine ZCS and ZnS. This exciting finding clearly shows an enormous potential in coupling Ti_3_C_2_ NPs with a wide variety of semiconductor photocatalysts/photoelectrodes.

## Discussion

This work demonstrates the great advantage of using modern theoretical tools for the design and synthesis of a novel MXene material, Ti_3_C_2_ NPs, as a highly active co-catalyst. On the basis of the theoretical predictions, we rationally employed the hydrothermal treatment to replace the –F terminations on Ti_3_C_2_ by –O/–OH terminations, and coupled the pretreated Ti_3_C_2_ with CdS to prepare a highly fused CdS/Ti_3_C_2_ composite photocatalyst. Remarkably, this composite photocatalyst exhibited both super high visible-light photocatalytic activity (14,342 μmol h^−1 ^g^−1^) and apparent quantum efficiency (40.1% at 420 nm), rendering it as one of the best noble-metal-free metal-sulfides photocatalysts. By combining the first-principle calculations and experimental methodology, we found that this unusual activity can be attributed to the synergetic effect of the highly efficient charge separation and migration from CdS to Ti_3_C_2_ NPs and the rapid H_2_ evolution on numerous –O terminations present on Ti_3_C_2_ NPs. Successful application of Ti_3_C_2_ NPs as an efficient co-catalyst on ZnS or ZCS excitingly confirms the versatile nature of this newly developed co-catalyst. This study opens a new area of utilizing this new generation of co-catalytic materials, MXene, to achieve highly efficient, steady and cost-effective solar water splitting based on semiconductor photocatalysts/photoelectrodes.

## Methods

### Materials synthesis

Ti_3_AlC_2_ (MAX phase: M_*n*+1_AX_*n*_, where M indicates early transition metal, A indicates III A or IV A group element, and X indicates C or N) was synthesized following the approach reported by Peng *et al*.^36^. Ti_3_C_2_-E was prepared by immersing Ti_3_AlC_2_ in HF solution. Ti_3_C_2_ NPs were fabricated by ultra-sonication of Ti_3_C_2_-E in de-ionized water. The detailed synthesis procedures of Ti_3_AlC_2_, Ti_3_C_2_-E and Ti_3_C_2_ NPs are described in Supplementary Methods. The CdS/Ti_3_C_2_ composites were fabricated by a one-step hydrothermal method summarized in Supplementary Methods. Pt–CdS was synthesized by *in situ* photo-deposition of 2.5 wt.% Pt on CT0 using H_2_PtCl_6_ aqueous solution. Pt NPs loaded CT0 (Pt–CdS-1) was synthesized by mixing 2.5 wt% Pt NPs with CT0 in ultra-sonication followed by washing with ethanol and dried at 60 °C. The morphology (Supplementary Fig. 25a) and photocatalytic activity (Supplementary Fig. 26) of Pt–CdS-1 are discussed in Supplementary Note 5. The above Pt NPs (Supplementary Fig. 27) was synthesized by a chemical-reduction method summarized in Supplementary Methods. NiS–CdS was synthesized following the previosuly reported method^37^ using CT0 as the substrate with 2.5 wt% loading of NiS. Ni–CdS was synthesized by *in situ* photo-deposition of 2.5 wt% Ni on CT0 using Ni(NO_3_)_2_ aqueous solution. MoS_2_–CdS was synthesized by the previously reported method^38^ using CT0 as the substrate with 2.5 wt% loading of MoS_2_. Ti_3_C_2_-5000 was synthesized following the preparation method of Ti_3_C_2_ NPs except that the final product was obtained by centrifugation at 5,000 r.p.m. CT2.5-5000 was prepared following the preparation method of CT2.5 except that Ti_3_C_2_-5000 was used instead of Ti_3_C_2_ NPs. HT-Ti_3_C_2_ NPs were synthesized following the hydrothermal method for preparation of CT2.5 except that no Cd(Ac)_2_ was added. CT2.5-A was acquired after the repeated photocatalytic reaction of CT2.5 for 28 h. Overall, 1 mol% NiS loaded CT0 (CN) was synthezised by following the previously reported method^39^. CNT2.5 was synthesized by a one-step hydrothermal method as summarized in Supplementary Methods. The phase structures (Supplementary Fig. 21b) and optical properties (Supplementary Fig. 21c) of CN and CNT2.5 are discussed in Supplementary Note 6. ZCS was synthesized by the previously reported method^39^. ZCS/Ti_3_C_2_ was synthesized by mechanical mixing of the as-synthesized ZCS with 1 wt.% Ti_3_C_2_ NPs. The phase structures (Supplementary Fig. 23b) and optical properties (Supplementary Fig. 23c) of ZCS and ZCS/Ti_3_C_2_ are discussed in Supplementary Note 7. ZnS was prepared by a hydrothermal approach as summarized in Supplementary Methods. ZnS/Ti_3_C_2_ was prepared by mechanical mixing of the as-synthesized ZnS with 1 wt.% Ti_3_C_2_ NPs. The phase structures (Supplementary Fig. 24b) and optical properties (Supplementary Fig. 24c) of ZnS and ZnS/Ti_3_C_2_ are discussed in Supplementary Note 8.

### Physicochemical characterization

XRD patterns were acquired on a powder X-ray diffractometer (Miniflex, Rigaku) using Cu-Kα radiation at 40 kV and 15 mA. SEM images and EDX spectra were collected on FEI Quanta 450 at an accelerating voltage of 10 kV. HAADF, TEM, HRTEM images and EDX were performed by utilizing a JEM-2100F electron microscope (JEOL, Japan). XPS measurements were conducted using an Axis Ultra (Kratos Analytical, UK) XPS spectrometer equipped with an Al Kα source (1,486.6 eV). The F/O atomic ratios in all the CdS/Ti_3_C_2_ composites were examined by XPS technique (Supplementary Fig. 28) and discussed in Supplementary Note 9. The Brunauer–Emmett–Teller specific surface areas (*S*_BET_) and pore volume (PV) of the samples were evaluated by N_2_ adsorption on a Tristar II 3020 gas adsorption apparatus (Micromeritics, USA). Ultraviolet-visible diffuse reflectance spectra were collected for the dry-pressed disk samples with an ultraviolet-visible spectrophotometer (UV2600, Shimadzu, Japan) using BaSO_4_ as the reflectance standard. PL spectra were recorded on a RF-5301PC spectrofluorophotometer (Shimadzu, Japan) at room temperature. Time-resolved PL decay curves were obtained on a FLS920 fluorescence lifetime spectrophotometer (Edinburgh Instruments, UK) under the excitation of 365 nm and probed at 460 nm. The actual chemical compositions of the as-synthesized samples were measured by ICP-AES using an Optima 4300 DV spectrometer (PerkinElmer) (Supplementary Table 2).

### Theoretical calculations

The DFT calculations were carried out by using the Vienna *ab initio* simulation package (VASP)^40^,^41^. The exchange-correlation interaction is described by generalized gradient approximation (GGA) with the Perdew–Burke–Ernzerhof (PBE) functional^42^. Van der Waals correction was applied in all calculations. The energy cutoff was set to 500 eV. The Brillouin zone was sampled by a Monkhorst-Pack 9 × 9 × 1 K-point grid. The fully relaxed lattice constants of Ti_3_C_2,_ O-terminated Ti_3_C_2_ and F-terminated Ti_3_C_2_ monolayers are 3.08, 3.01 and 3.02 Å respectively. The models of Ti_3_C_2_, O-terminated Ti_3_C_2_ or F-terminated Ti_3_C_2_ in 2 × 2 × 1 supercells with a *k*-point of 5 × 5 × 1 grid in reciprocal space are used to identify the HER activity sites. HSE06 calculations^43^,^44^ employing VASP are performed to get the exact band structures. The band gap is zero. The further calculation details of the Gibbs free energy of the absorption of atomic H, the Fermi level positions and the surface Pourbaix diagrams can be found in Supplementary Methods. The surface Pourbaix diagram (Supplementary Fig. 29) of Ti_3_C_2_ is analysed and discussed in Supplementary Note 10. The excellent conductivity of O-terminated Ti_3_C_2_ at different H coverages (Supplementary Fig. 30) is confirmed in Supplementary Note 11.

### Photocatalytic H_2_-production test

The experimental measurements of photocatalytic H_2_ production were carried out in a 100 ml Pyrex flask (openings sealed with silicone rubber septum) at room temperature and atmospheric pressure. A 300 W Xenon arc lamp with an ultraviolet-cutoff filter (*λ*≥420 nm) was utilized as a visible-light source to trigger the photocatalytic reaction. The focused intensity on the flask was ca. 80 mW cm^−2^. Typically, 20 mg of the photocatalyst was suspended by constant stirring in 80 ml of mixed aqueous solution containing 20 ml of lactic acid (88 vol%) and 60 ml of water. Before irradiation, the suspension was purged with Argon for 0.5 h to remove any dissolved air and keep the reaction system under anaerobic conditions. Next, 0.2 ml gas was intermittently sampled through the septum, and H_2_ content was analysed by gas chromatograph (Clarus 480, PerkinElmer, USA, TCD, Ar as a carrier gas and 5 Å molecular sieve column). Before the experiment, all glassware was rinsed carefully with de-ionized water. The apparent quantum efficiency (QE) was measured under the identical photocatalytic reactions. Four low power 420-nm LEDs (3 W, Shenzhen LAMPLIC Science Co Ltd. China) were employed as the light sources to trigger the photocatalytic reactions. The focused intensity for every 420-nm LED was ca. 6 mW cm^−2^. The QE was calculated according to the following equation (1):


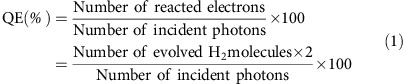


### Electrochemical and photoelectrochemical measurements

EIS measurements were performed on an electrochemical analyser (CHI650D instruments) in a standard three-electrode system utilizing the synthesized samples as the working electrodes, Ag/AgCl (saturated KCl) as a reference electrode, and a Pt wire as the counter electrode. The polarization curves were recorded in the above-mentioned three-electrode system and the bias sweep range was from −1.5 to −0.8 V versus Ag/AgCl with a step size of 0.005 V. 0.5 M Na_2_SO_4_ aqueous solution was utilized as the electrolyte. The Mott-Schottky plots were also measured using the same three-electrode system over an alternating current (AC) frequency of 1,200 Hz in 0.5 M Na_2_SO_4_ aqueous solution. The EIS were recorded over a range from 1 to 2 × 10^5^ Hz with an AC amplitude of 0.02 V. 0.5 M potassium phosphate buffer solution was used as the electrolyte. Photocurrent was measured in the same three-electrode system. A 300 W Xenon light with an ultraviolet-cutoff filter (*λ*≥420 nm) was applied as the light source. 0.2 M Na_2_S and 0.04 M Na_2_SO_3_ mixed aqueous solution was used as the electrolyte. The working electrodes were synthesized as follows: 0.1 g sample and 0.03 g polyethylene glycol (PEG; molecular weight: 20,000) were ground with 0.5 ml of ethanol to make a slurry. Then the slurry was coated onto a 2 cm × 1.5 cm FTO glass electrode by the doctor blade approach. The obtained electrode was dried in an oven and heated at 623 K for 0.5 h under flowing N_2_. All working electrodes studied were kept at a similar film thickness of about 10–11 μm.

### Data availability

The data that support the findings of this study are available from the corresponding author on request.

## Additional information

**How to cite this article:** Ran, J. *et al*. Ti_3_C_2_ MXene co-catalyst on metal sulfide photo-absorbers for enhanced visible-light photocatalytic hydrogen production. *Nat. Commun.* **8,** 13907 doi: 10.1038/ncomms13907 (2017).

**Publisher's note:** Springer Nature remains neutral with regard to jurisdictional claims in published maps and institutional affiliations.

## Supplementary Material

Supplementary Information Supplementary figures, supplementary tables, supplementary notes, supplementary methods and supplementary references
