# Supplementary material for: Ti3C2 MXene co-catalyst on metal sulfide photo-absorbers for enhanced visible-light photocatalytic hydrogen production
Source: Nat Commun. 2017 Jan 3;8:13907. doi: 10.1038/ncomms13907 (PMC5512649; doi:10.1038/ncomms13907)
Supplement: Supplementary Information — Supplementary figures, supplementary tables, supplementary notes, supplementary methods and supplementary references [file ncomms13907-s1.pdf]

Supplementary Figures

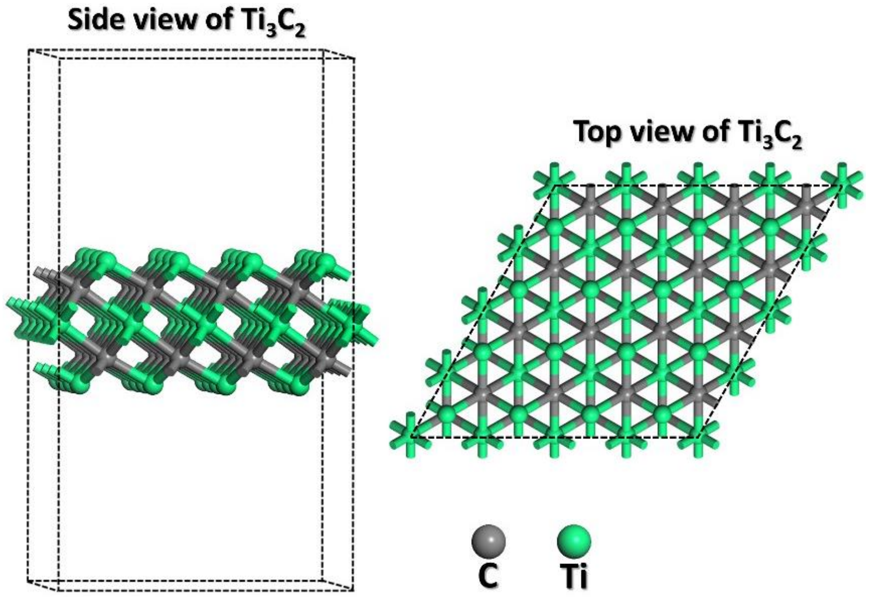

Supplementary Figure 1. The side and top views of structural model for a 4×4×1 Ti_3C_2 supercell.

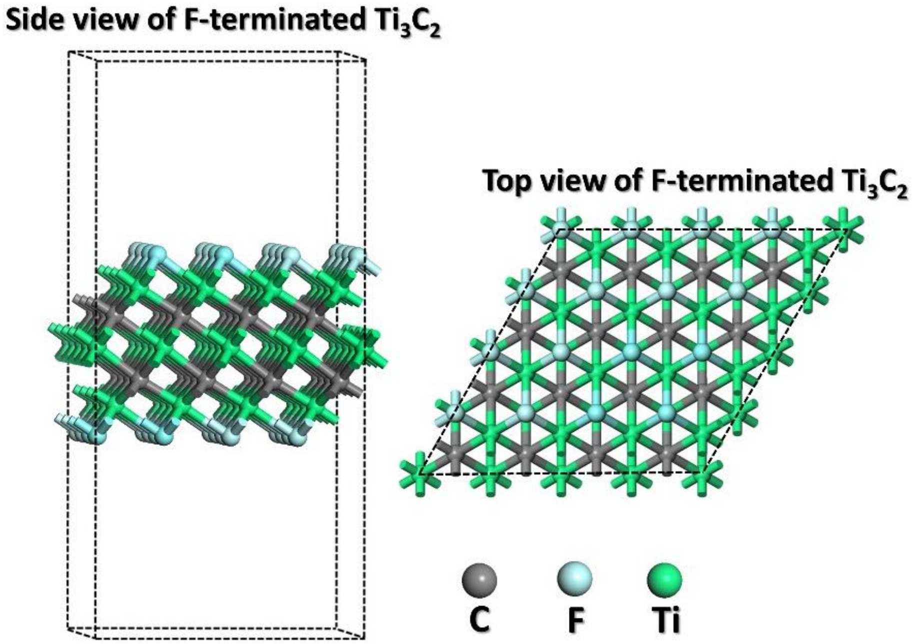

Supplementary Figure 2. The side and top views of structural model for a 4×4×1 F-terminated Ti_3C_2 supercell.

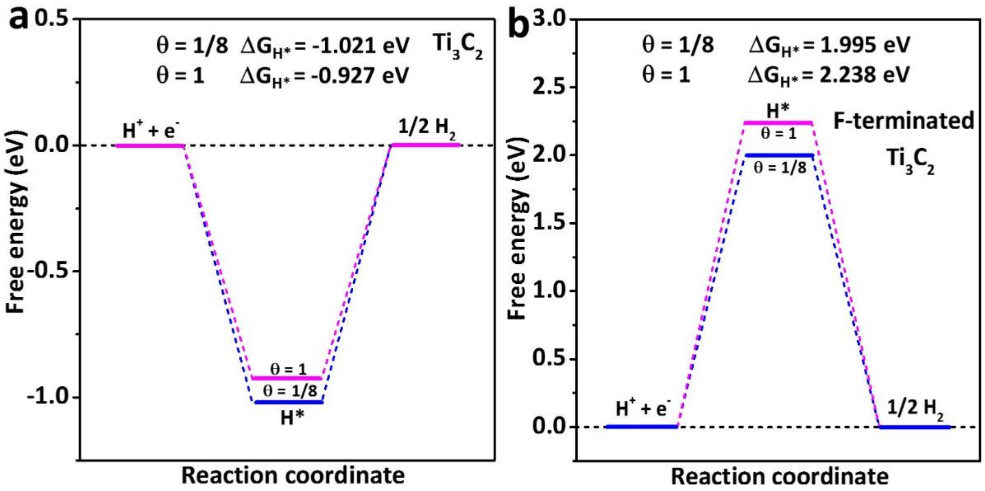

Supplementary Figure 3. (**a**) The calculated free-energy diagram of HER at the equilibrium potential (U = 0 V) on the surface of a 2×2×1 Ti_3C_2 supercell and (**b**) a 2×2×1 F-terminated Ti_3C_2 supercell under different H* coverage conditions (1/8 and 1). The insets in (**a**) and (**b**) show the calculated Gibbs free energies for H adsorption on the surface of a 2×2×1 Ti_3C_2 supercell and a 2×2×1 F-terminated Ti_3C_2 supercell with different H* coverages, respectively.

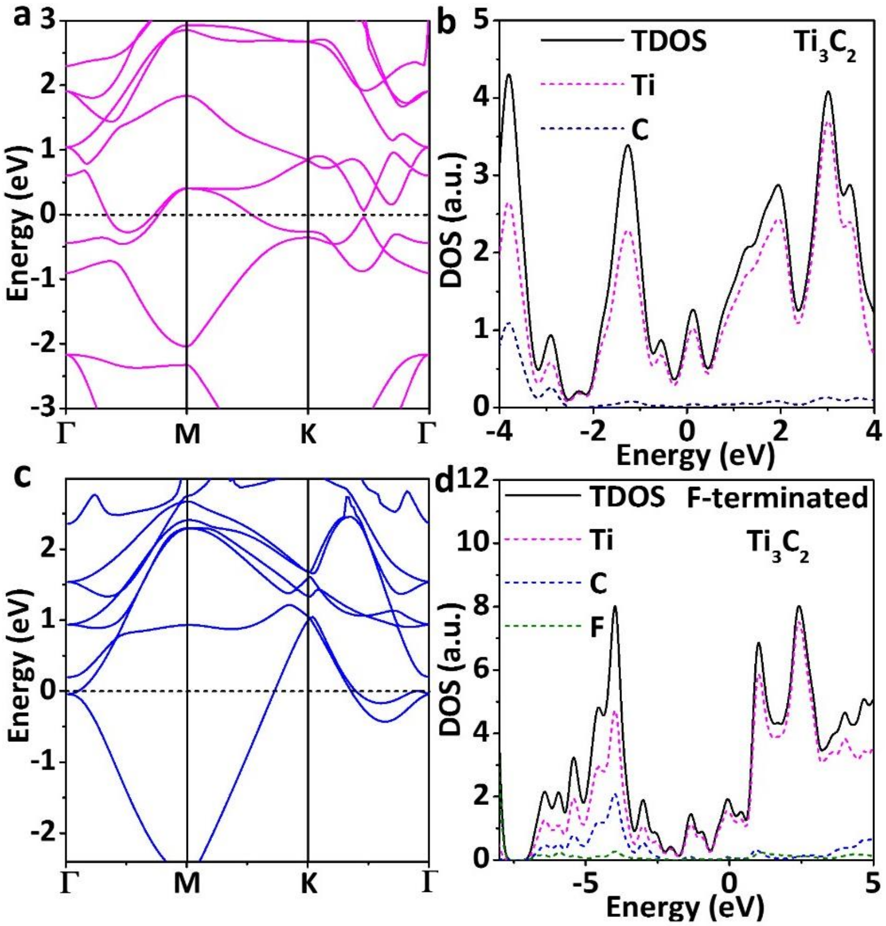

Supplementary Figure 4. (**a**) The electronic band structure and (**b**) the total density of states (TDOS) and partial density of states (PDOS) for pure Ti_3C_2. (**c**) The electronic band structure and (**d**) the TDOS and PDOS for F-terminated Ti_3C_2.

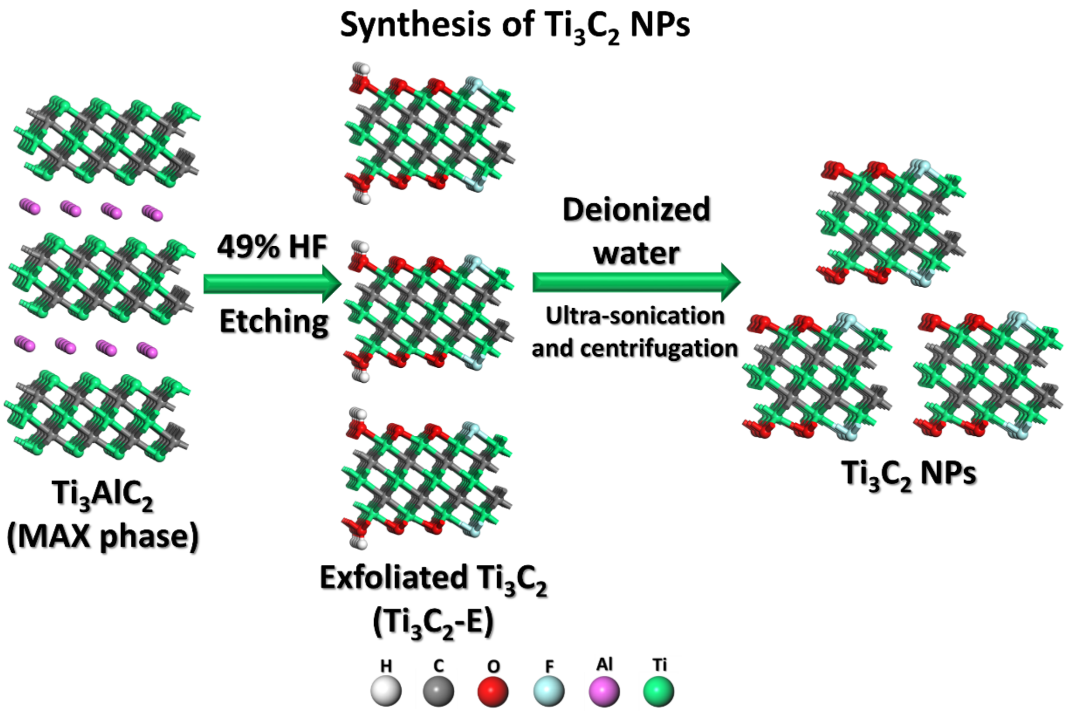

Supplementary Figure 5. Synthesis procedure of Ti_3C_2 NPs.

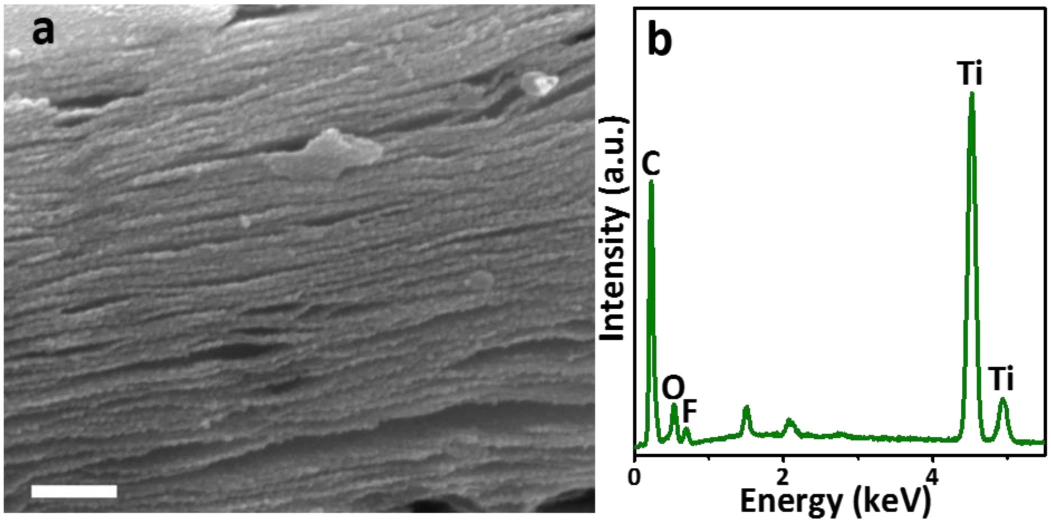

Supplementary Figure 6. (**a**) SEM image and (**b**) the corresponding EDX pattern of Ti_3C_2-E. Scale bar, 500 nm (**a**).

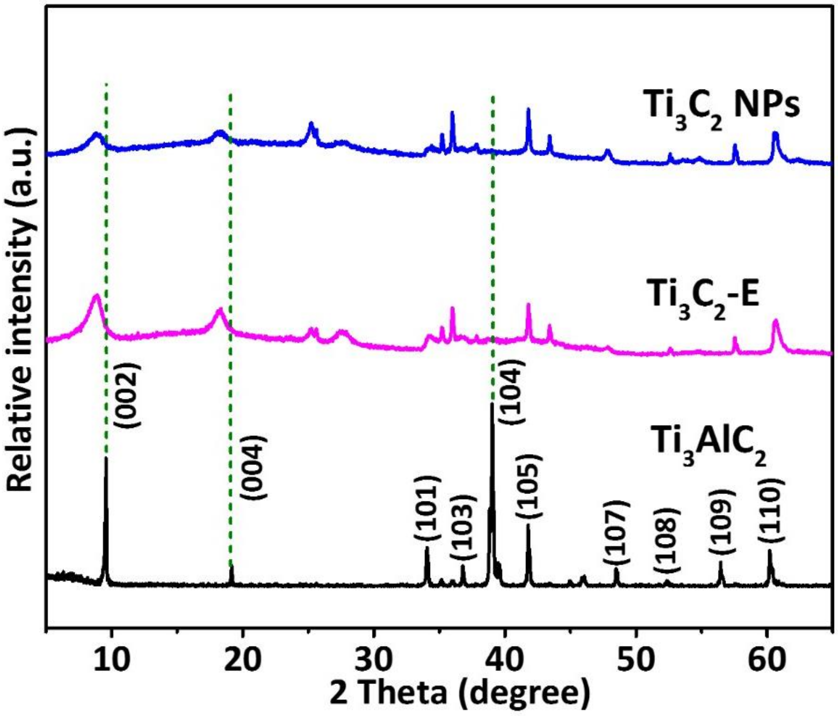

Supplementary Figure 7. XRD patterns of Ti_3AlC_2, Ti_3C_2-E and Ti_3C_2 NPs.

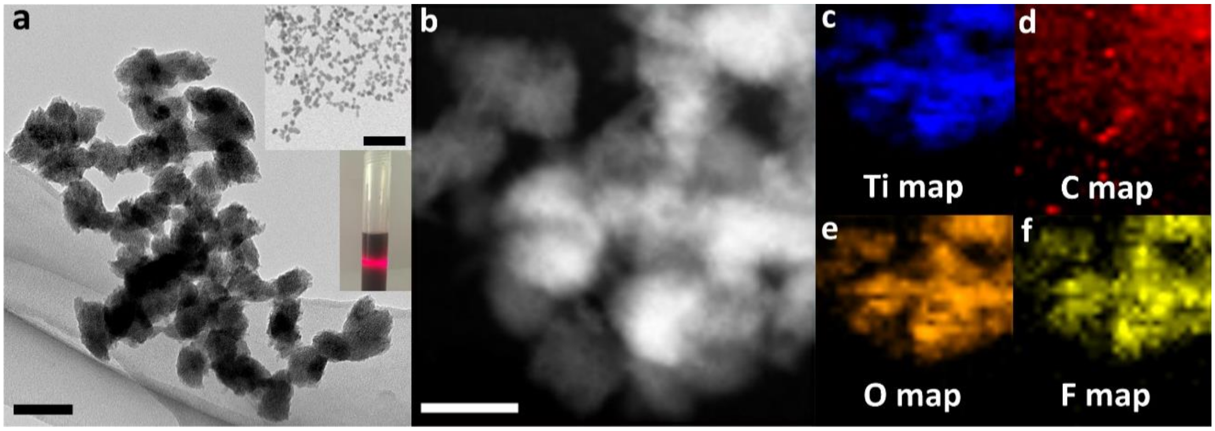

Supplementary Figure 8. (**a**) High-magnification TEM image of Ti_3C_2 NPs with inset showing the low-magnification TEM image of Ti_3C_2 NPs and the Tyndall effect of Ti_3C_2 dispersion. (**b**) HAADF image of Ti_3C_2 NPs and its corresponding EDX elemental mapping images of (**c**) Ti, (**d**) C, (**e**) O and (**f**) F. Scale bars, 100 nm (**a**), 500 nm inset in (**a**) and 50 nm (**b**).

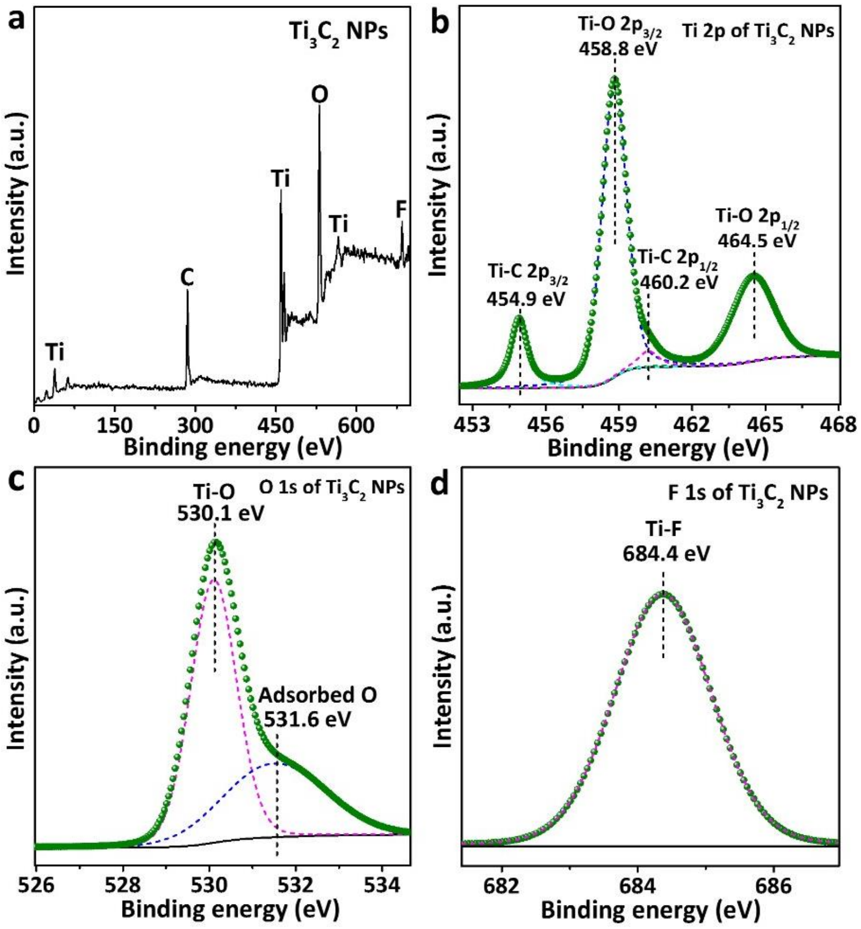

Supplementary Figure 9. (**a**) XPS survey spectrum of Ti_3C_2 NPs. High-resolution XPS spectra of (**b**) Ti 2p, (**c**) O 1s and (**d**) F 1s for Ti_3C_2 NPs.

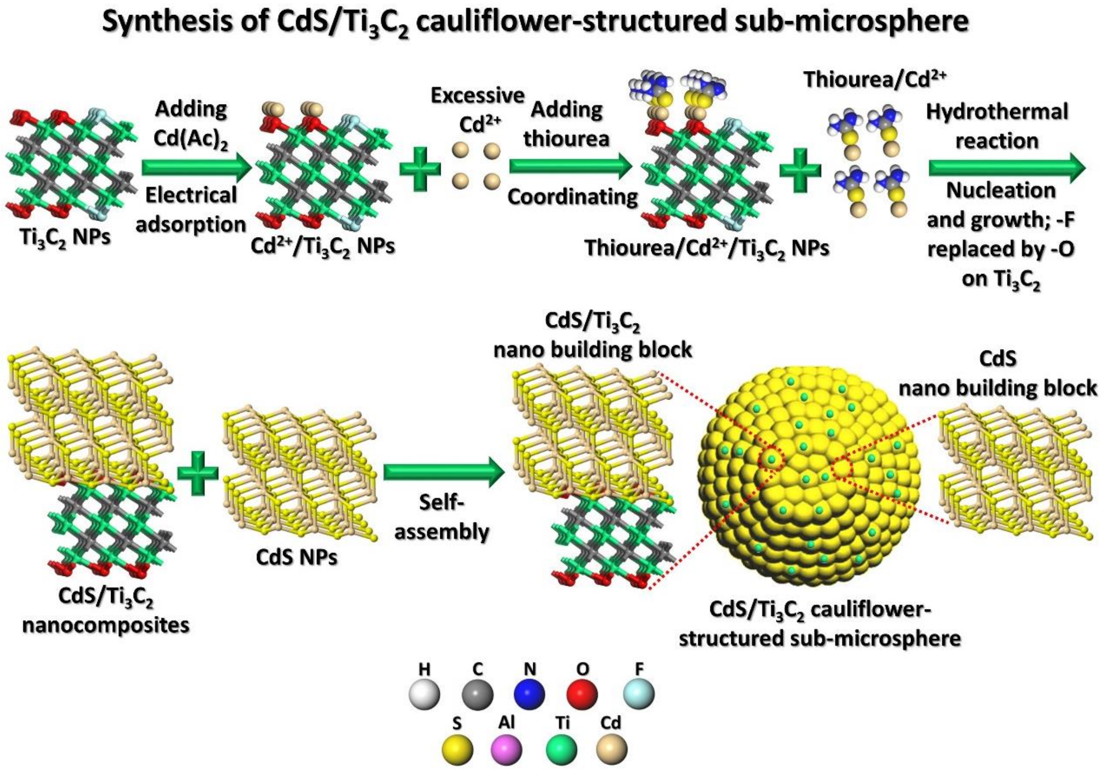

Supplementary Figure 10. Synthesis procedure of CdS/Ti_3C_2 cauliflower-structured sub-microsphere (SMS).

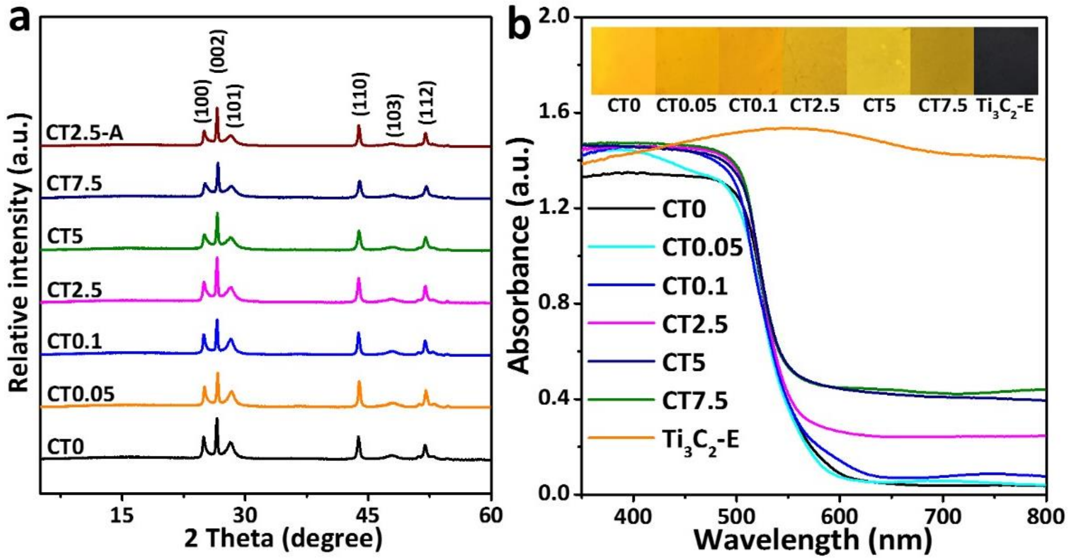

Supplementary Figure 11. (**a**) XRD patterns of CT0, CT0.05, CT0.1, CT2.5, CT5, CT7.5 and CT2.5-A. (**b**) UV-Vis diffuse reflectance spectra of CT0, CT0.05, CT0.1, CT2.5, CT5, CT7.5 and Ti_3C_2-E. The inset in (**b**) shows the colors of the above samples.

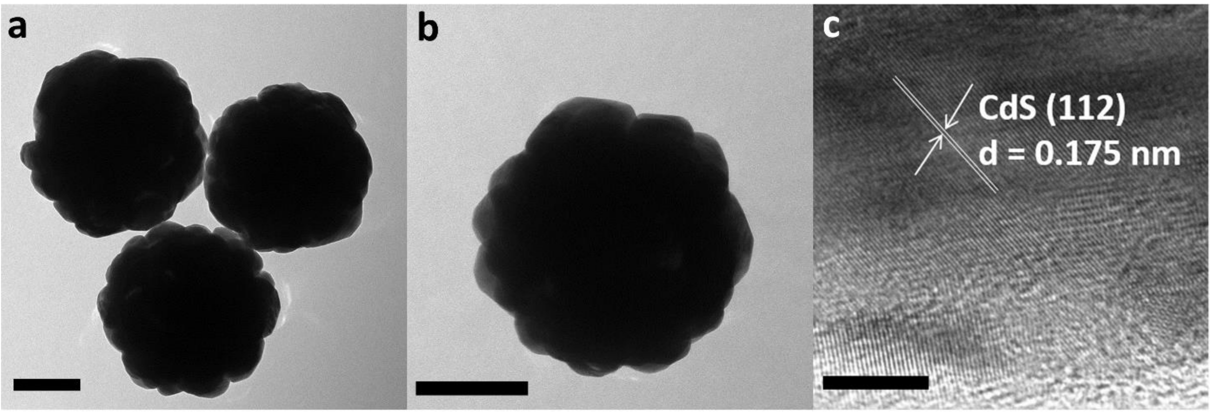

Supplementary Figure 12. (**a, b**) TEM images and (**c**) HRTEM image of CT0. Scale bars, 200 nm (**a, b**), 5 nm (**c**).

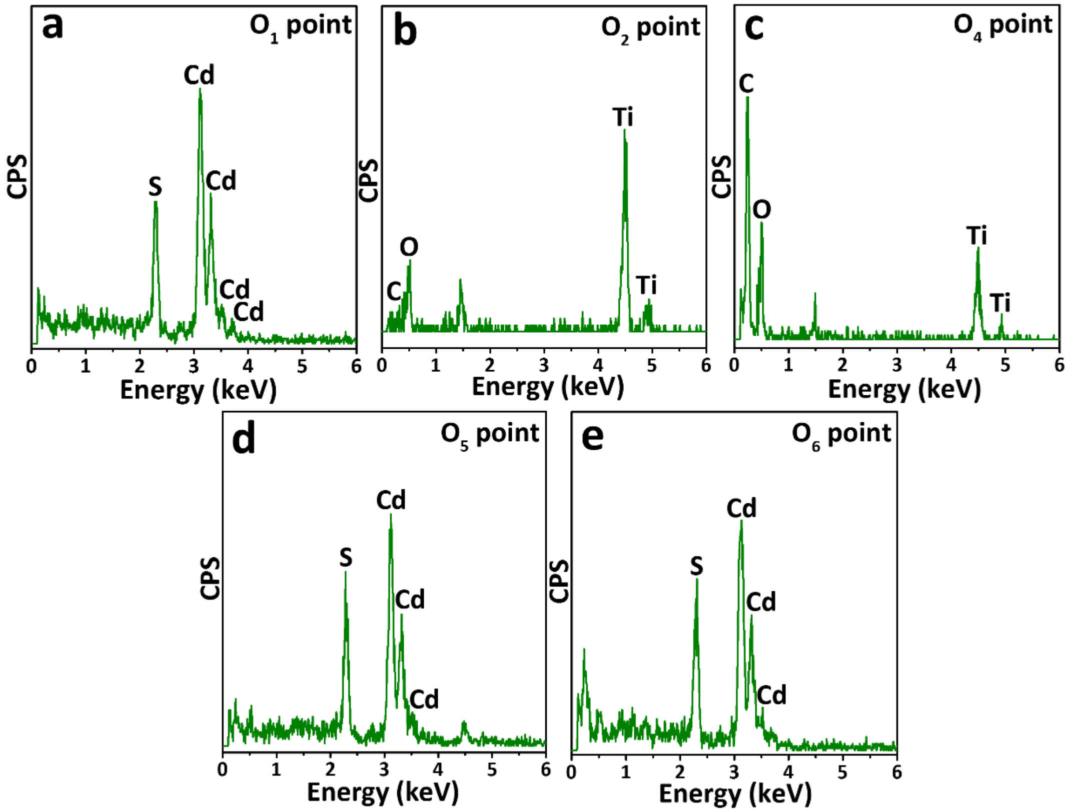

Supplementary Figure 13. The EDX spectra at (**a**) O_1, (**b**) O_2, (**c**) O_4, (**d**) O_5 and (**e**) O_6 points in Fig. 2a.

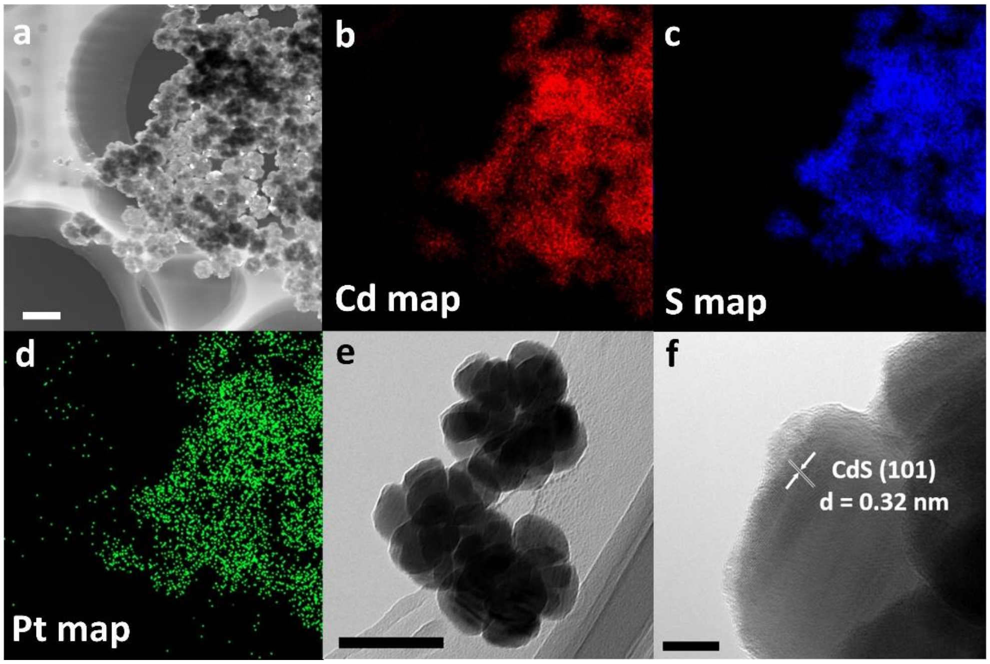

Supplementary Figure 14. (**a**) A typical HAADF image of Pt-CdS and its corresponding EDX elemental mapping images of (**b**) Cd, (**c**) S and (**d**) Pt. The typical (**e**) TEM and (**f**) HRTEM images of Pt-CdS. Scale bars, 200 nm (**a**), 100 nm (**e**) and 10 nm (**f**).

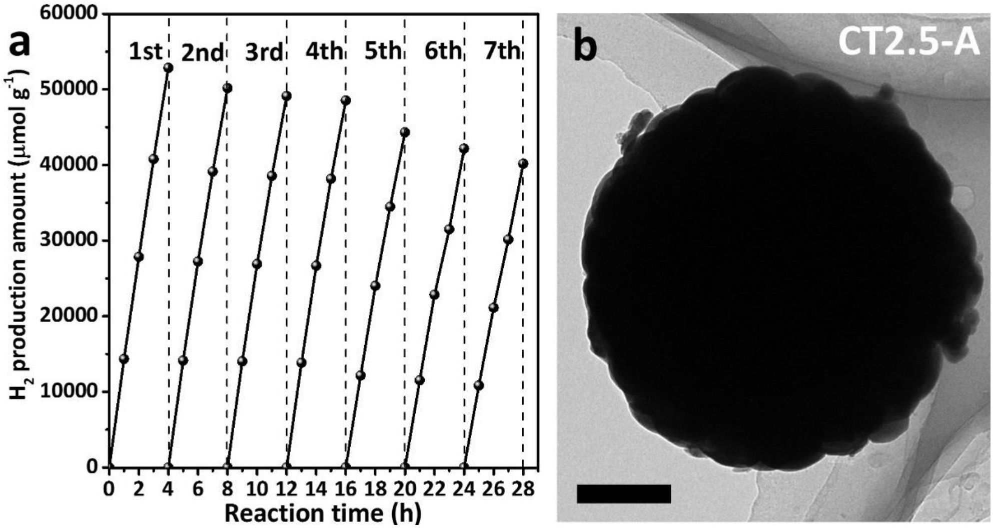

Supplementary Figure 15. (**a**) Time course of photocatalytic H_2 production over CT2.5; every 4 hours the reaction system was purged with Ar for 30 min to remove H_2. (**b**) A typical TEM image of CT2.5-A. Scale bar, 200 nm (**b**).

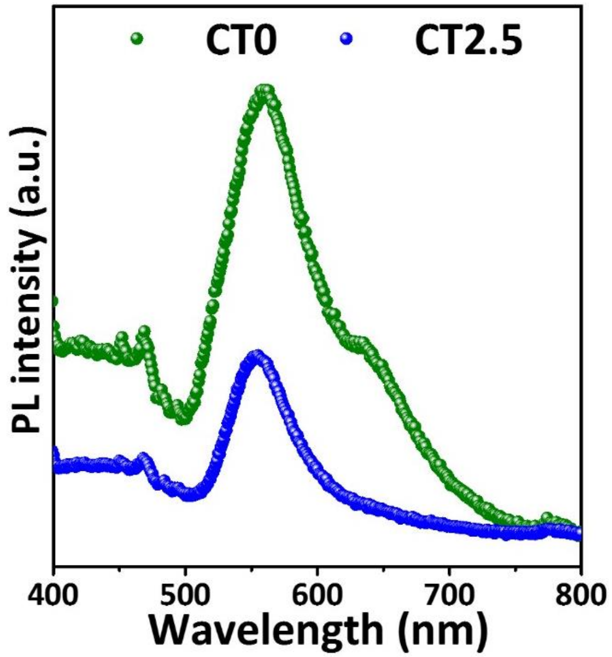

Supplementary Figure 16. PL spectra of CT0 and CT2.5.

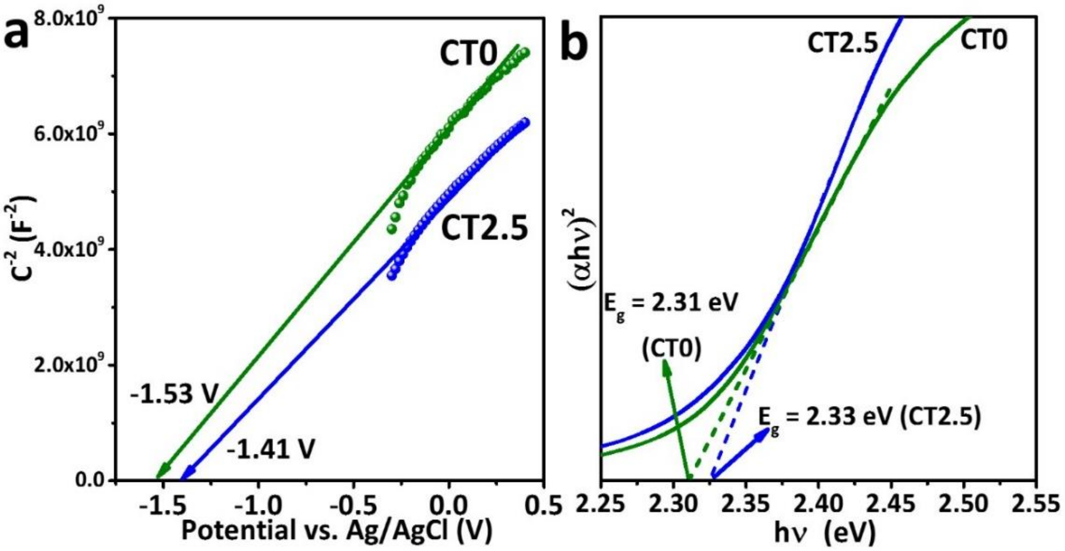

Supplementary Figure 17. (**a**) Mott-Schottky plots of CT0 and CT2.5 electrodes in 0.5 M Na_2SO_4 aqueous solution. (**b**) Tauc plots of CT0 and CT2.5.

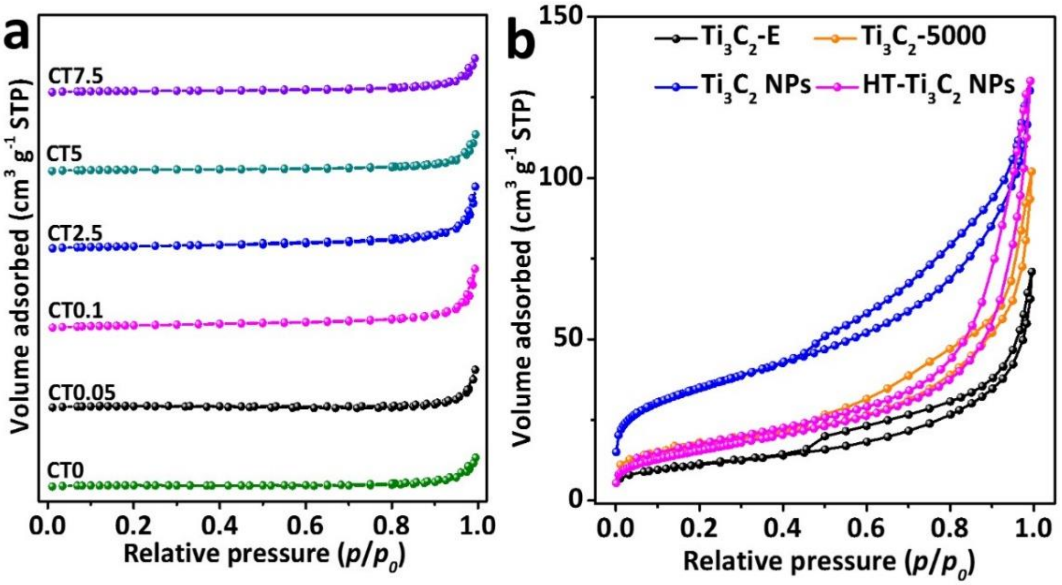

Supplementary Figure 18. (**a**) N_2 sorption isotherms of CT0, CT0.05, CT0.1, CT2.5, CT5 and CT7.5. (**b**) N_2 sorption isotherms of Ti_3C_2-E, Ti_3C_2-5000, Ti_3C_2 NPs and HT-Ti_3C_2 NPs.

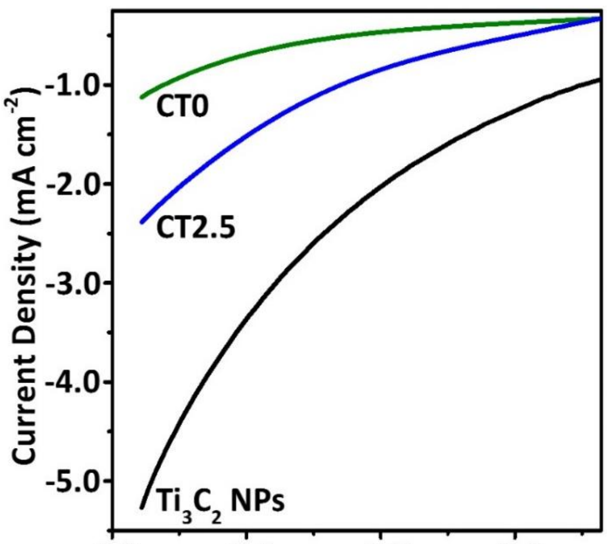

Supplementary Figure 19. Polarization curves of CT0, CT2.5 and Ti_3C_2 NPs electrodes in 0.5 M Na_2SO_4 aqueous solution.

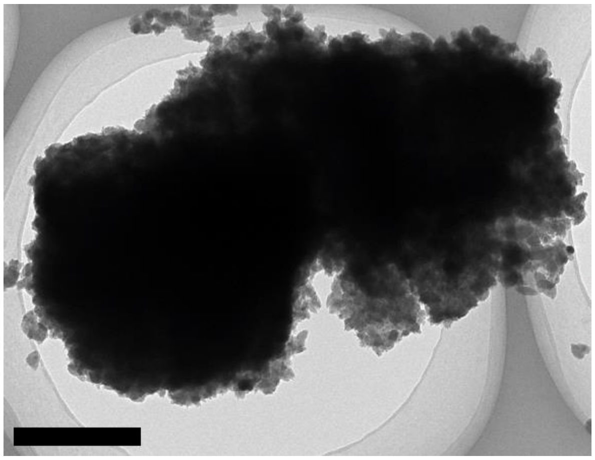

Supplementary Figure 20. TEM image of Ti_3C_2-5000. Scale bar, 500 nm.

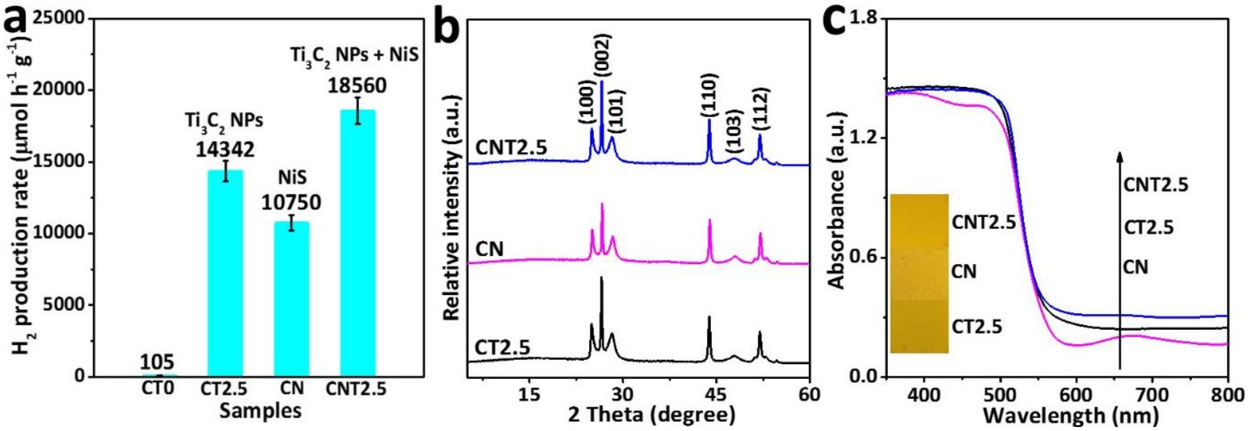

Supplementary Figure 21. (**a**) A comparison of the photocatalytic H_2-production activities of CT0, CT2.5, CN and CNT2.5, using 18 vol.% lactic acid aqueous solution as a sacrificial reagent under visible-light irradiation ($\lambda \geq 420$ nm, 300 W Xe lamp). The error bars are defined as s. d. (**b**) XRD patterns of CT2.5, CN and CNT2.5. (**c**) UV-Vis diffuse reflectance spectra of CT2.5, CN and CNT2.5. The inset shows the colors of the above samples.

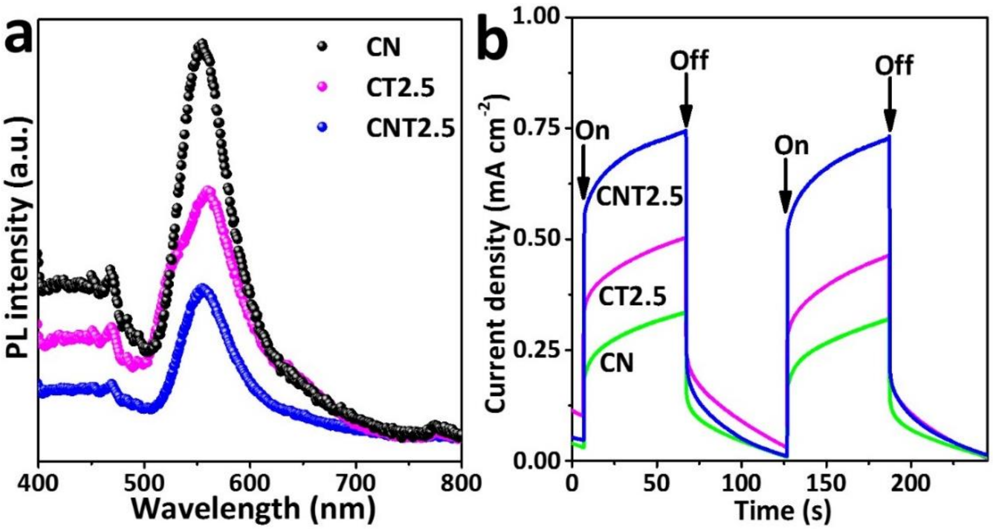

Supplementary Figure 22. (**a**) PL spectra of CT2.5, CN and CNT2.5. (**b**) TPC responses of CT2.5, CN, and CNT2.5 electrodes in 0.2 M Na_2S + 0.04 M Na_2SO_3 mixed aqueous solution under visible-light irradiation.

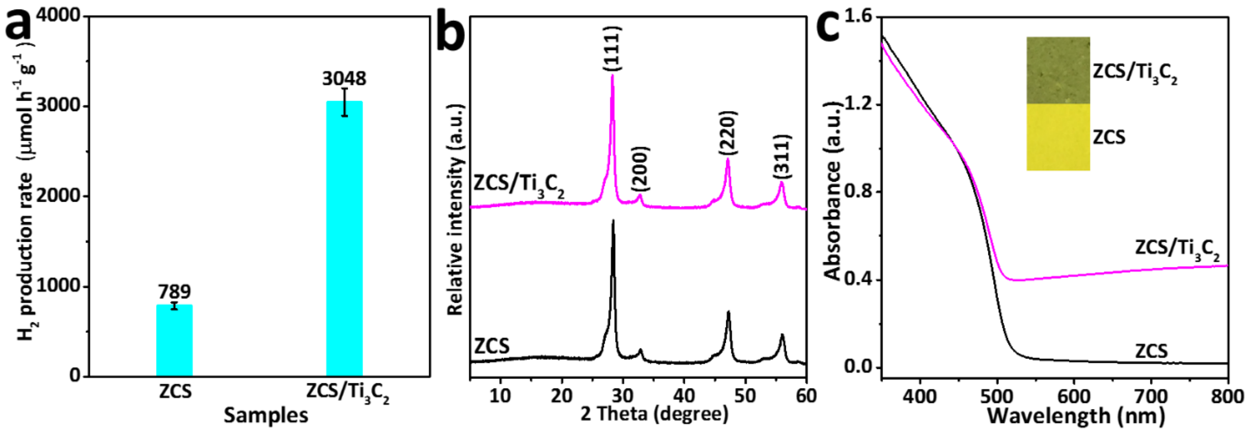

Supplementary Figure 23. (**a**) A comparison of the photocatalytic H_2-production activities of ZCS and ZCS/Ti_3C_2 using 18 vol.% lactic acid aqueous solution as a sacrificial reagent under visible-light irradiation ($\lambda \geq 420$ nm, 300 W Xe lamp). The error bars are defined as s. d. (**b**) XRD patterns of ZCS and ZCS/Ti_3C_2. (**c**) UV-Vis diffuse reflectance spectra of ZCS and ZCS/Ti_3C_2. The inset shows the colors of the above samples.

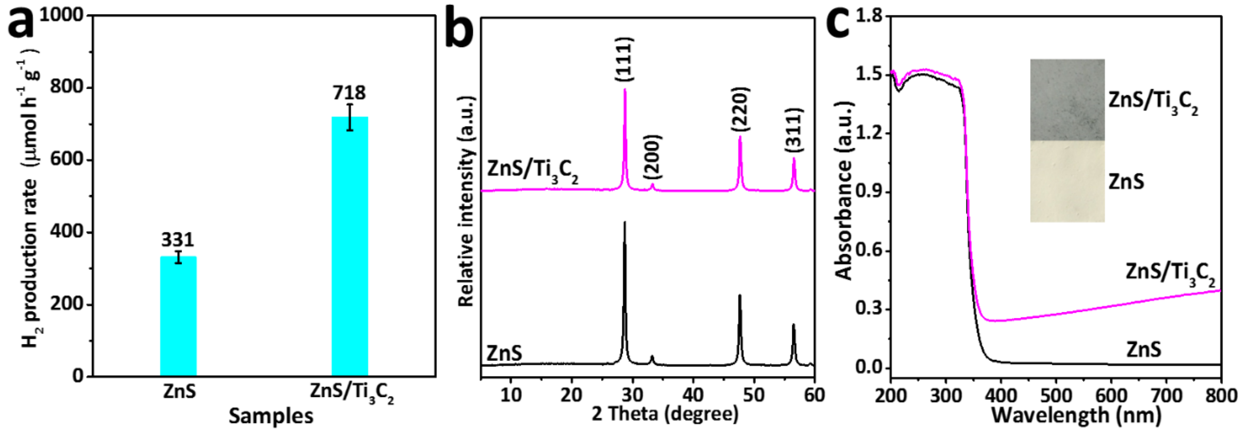

Supplementary Figure 24. (**a**) A comparison of the photocatalytic H_2-production activities of ZnS and ZnS/Ti_3C_2 using 18 vol.% lactic acid aqueous solution as a sacrificial reagent under light irradiation (300 W Xe lamp). The error bars are defined as s. d. (**b**) XRD patterns of ZnS and ZnS/Ti_3C_2. (**c**) UV-Vis diffuse reflectance spectra of ZnS and ZnS/Ti_3C_2. The inset shows the colors of the above samples.

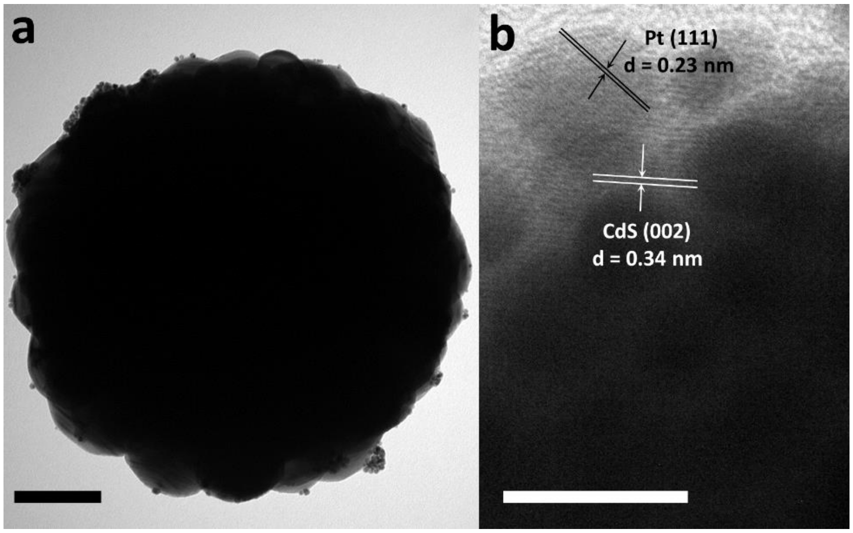

Supplementary Figure 25. The (**a**) TEM and (**b**) HRTEM images of Pt-CdS-1. Scale bars, 100 nm (**a**) and 10 nm (**b**).

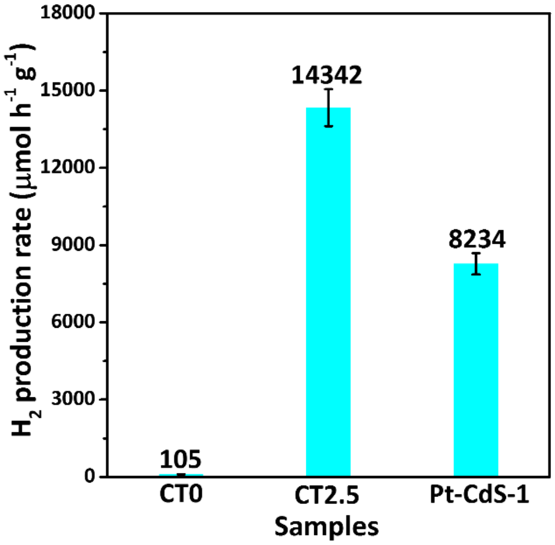

Supplementary Figure 26. A comparison of the photocatalytic H_2-production activities of CT0, CT2.5 and Pt-CdS-1 using 18 vol.% lactic acid aqueous solution as a sacrificial reagent under visible-light irradiation ($\lambda \geq 420$ nm, 300 W Xe lamp). The error bars are defined as s. d.

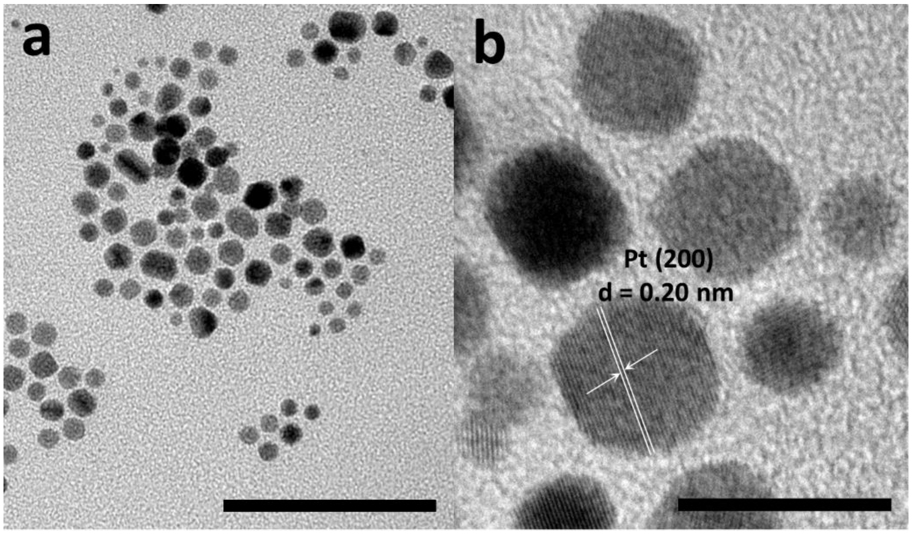

Supplementary Figure 27. The (**a**) TEM and (**b**) HRTEM images of Pt NPs synthesized by the chemical reduction method. Scale bars, 50 nm (**a**) and 10 nm (**b**).

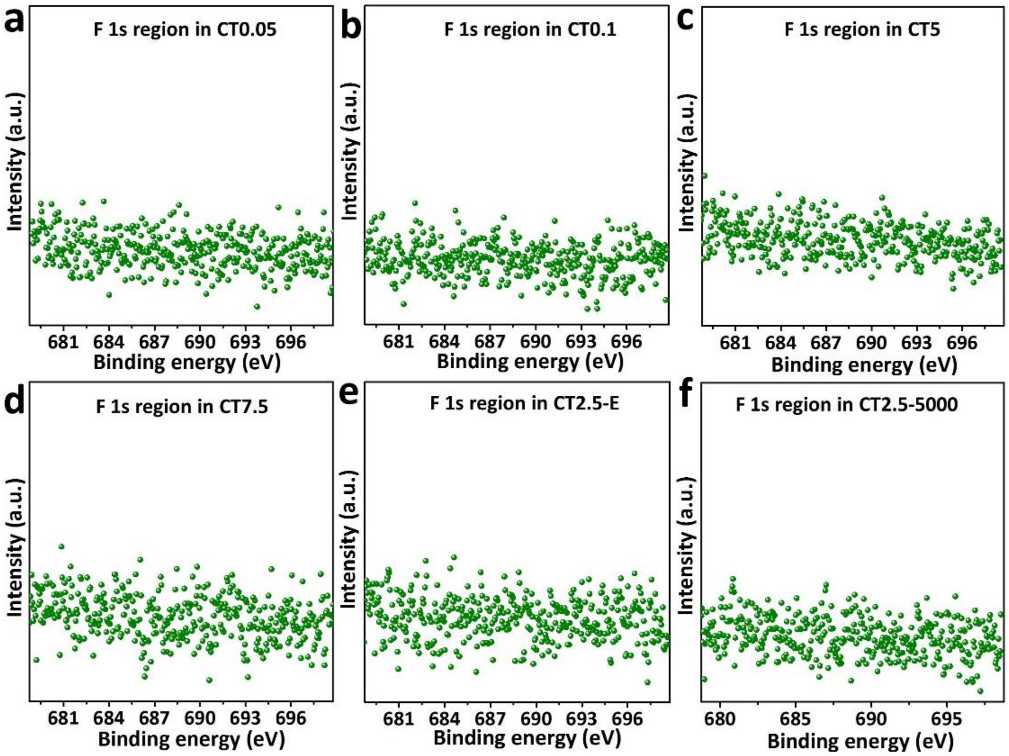

Supplementary Figure 28. The high-resolution XPS spectra of F1s for (**a**) CT0.05, (**b**) CT0.1, (**c**) CT5, (**d**) CT7.5, (**e**) CT2.5-E and (**f**) CT2.5-5000.

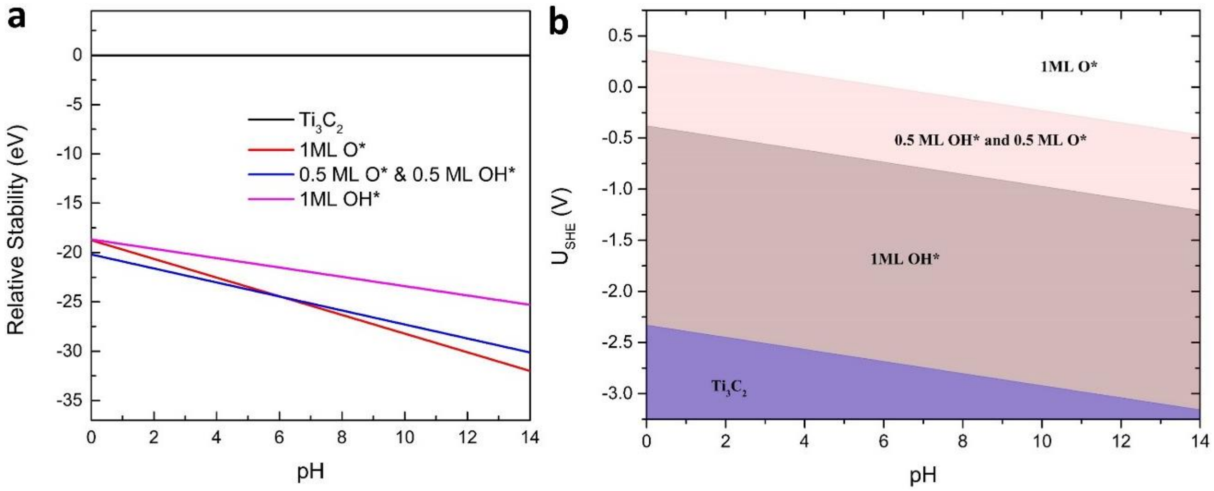

Supplementary Figure 29. (**a**) Stability of O* and OH* on Ti_3C_2 at $U_{SHE} = 0$ including alkaline and acidic dissolution. (**b**) Surface Pourbaix diagram for Ti_3C_2.

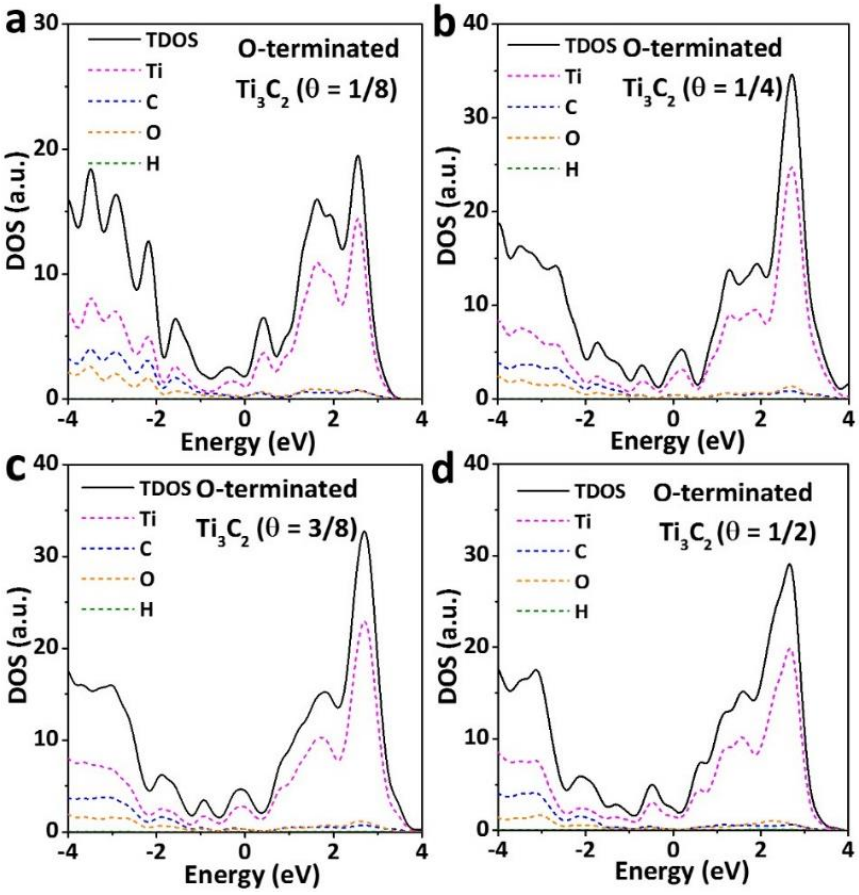

Supplementary Figure 30. The density of states for O-terminated Ti_3C_2 at H coverages (θ) of (**a**) 1/8 , (**b**) 1/4, (**c**) 3/8 and (**d**) 1/2.

Supplementary Tables

Supplementary Table 1. The H adsorption energy (ΔG_{H^*}) on O-terminated Ti_3C_2 at different H* coverages.

H* coverage θ	H adsorption energy ΔG_{H^*} (eV)
1/8	-0.316
1/4	-0.254
3/8	0.0468
1/2	-0.00283
5/8	0.470
3/4	0.527

Supplementary Table 2. Physicochemical Properties of CT0, CT0.05, CT0.1, CT2.5, CT5 and CT7.5.

Samples	Ti_3C_2 (wt.%) (ICP-AES)	S_{BET} (m^2 g^{-1})	PV[a] (cm^3 g^{-1})	ACS[b] (nm)	H_2 production rate (μmol h^{-1} g^{-1})
CT0	0	3.8	0.01	53	105
CT0.05	0.03	4.2	0.01	54	993
CT0.1	0.09	7.1	0.02	55	1278
CT2.5	1.89	7.2	0.02	52	14342
CT5	4.13	3.8	0.01	55	3377
CT7.5	6.79	3.7	0.01	49	2707

[a] PV: Pore volume, [b] ACS: Average crystallite size.

Supplementary Table 3. Comparison of the photocatalytic H_2-production activities for the representative binary CdS-based photocatalysts loaded with different non-noble metal co-catalysts.

Photocatalyst	*Amount of photocatalyst (mg)*	*Co-catalyst*	*Loading method*	*Optimum loading*	*Enhancement factor*	*Photocatalytic H_2-production activity (μmol h^{-1} g^{-1})*	*Apparent quantum efficiency*	*Reference*
CT2.5	20	Ti_3C_2 NPs	Hydrothermal	2.5 wt.%	136.6	14,342	40.1% at 420 nm	This work
Ni/CdS	100	Ni	Solution mixing	4 wt.%	N/A	25,848	26.8% at 420 nm	[1]
NiO_x/CdS	100	NiO_x	Photo-deposition	1 mol.%	117	5,908	N/A	[2]
$Ni(OH)_2$/CdS	50	$Ni(OH)_2$	Liquid precipitation	23 mol.%	145	5,084	28% at 420 nm	[3]
NiS/CdS	50	NiS	Hydrothermal	5 mol.%	20.6	1,131	N/A	[4]
Ni_2P/CdS	1	Ni_2P	Grinding	5 wt.%	N/A	143,600	14.3% at 420 nm	[5]
Co_3O_4/CdS	50	Co_3O_4	Hydrothermal	3 mol.%	33	236	N/A	[6]
CoP/CdS	1	CoP	Grinding	5 wt.%	N/A	254,000	25.1% at 420 nm	[5]
Graphene oxide/CdS	100	Graphene oxide	Electrostatic assembly	5 wt.%	1.3	3,140	4.8% at 420 nm	[7]
Carbon nanotube/CdS	100	Carbon nanotube	Hydrothermal	3 wt.%	3.7	520	N/A	[8]
MoS_2/CdS	50	MoS_2	Solvothermal	2.5 mol.%	N/A	11,026	31.8% at 420 nm	[9]
WS_2/CdS	10	WS_2	Hot-injection	11 mol.%	16.7	1,984	N/A	[10]

Supplementary Table 4. BET surface area and pore volume of Ti_3C_2-E, Ti_3C_2-5000, Ti_3C_2 NPs, and HT-Ti_3C_2 NPs.

Samples	BET surface area ($m^2\ g^{-1}$)	Pore volume ($cm^3\ g^{-1}$)
Ti_3C_2-E	39.6	0.08
Ti_3C_2-5000	61.6	0.11
Ti_3C_2 NPs	120.1	0.20
HT-Ti_3C_2 NPs	56.7	0.10

Supplementary Notes

Supplementary Note 1. Analysis of the XRD patterns of Ti_3AlC_2, Ti_3C_2-E and Ti_3C_2 NPs

As shown in Supplementary Fig. 7, the XRD patterns of Ti_3AlC_2, Ti_3C_2-E and Ti_3C_2 NPs are consistent with the literature[11,12]. After ultra-sonication treatment, Ti_3C_2 NPs show a decrease in the intensities of (002) and (004) peaks, in agreement with the dimension change from 3D Ti_3C_2-E to 0D Ti_3C_2 NPs.

Supplementary Note 2. Analysis of the morphology and chemical composition of Pt-CdS

The EDX elemental mapping images of Cd, S and Pt (Supplementary Fig. 14b-d) are consistent with the HAADF image of Pt-CdS (Supplementary Fig. 14a), indicating that Pt is homogeneously dispersed on the surface of CdS. However, no obvious Pt NPs are observed on the surface of CdS in both TEM and HRTEM images (Supplementary Fig. 14e and f), suggesting that Pt is decorated on CdS in the form of ultra-small clusters. Moreover, the petal-like assembly of CdS NPs (Supplementary Fig. 14e) is due to the disassembly of the CdS SMSs (Supplementary Fig. 12a and b). This disassembly is caused by the deposition of a Pt layer on the surface of the CdS NPs, which consequently weakens the combination between CdS NPs.

Supplementary Note 3. Explanation of the hydrogen adsorption energy (ΔG_{H^*}) change on Ti_3C_2

In general, the change in ΔG_{H^*} is continuous from one coverage to the next. In this work, O terminations on both the top and bottom sides of Ti_3C_2 can function as the active sites for HER. The ΔG_{H^*} at $\theta = 3/8$ refers to the free energy of the second H adsorbing on the top side of the 2×2×1 O-terminated Ti_3C_2 model. The ΔG_{H^*} at $\theta = 1/2$ refers to the free energy of the second H adsorbing on the bottom side of the 2×2×1 O-terminated Ti_3C_2 model. Both of them refer to the 1/2 H surface coverage. However, at $\theta = 1/2$ (two H on both the top and bottom sides, respectively), the adsorption system possesses higher symmetry, which decreases the H binding energy. Therefore, $|\Delta G_{H^*}|$ ($\theta = 1/2$) < $|\Delta G_{H^*}|$ ($\theta = 3/8$) as shown in Supplementary Table 1.

Supplementary Note 4. Confirmation of the electron transfer from CdS to Ti_3C_2 in CT2.5

The Mott-Schottky plot (Supplementary Fig. 17a) suggests that the flat band potential of CT0 (pure CdS) is -1.53 V *vs.* Ag/AgCl, which corresponds to -0.91 V *vs.* SHE. Thus, the Fermi level of pure CdS is near -0.91 V *vs.* SHE. On the other hand, the Fermi level of O-terminated Ti_3C_2 is calculated to be 1.88 V *vs.* SHE. Hence, the electrons should migrate from CdS to Ti_3C_2 upon their combination. This is evidenced by the more positive flat band potential of CT2.5 (-0.79 V *vs.* SHE) compared with that of CT0, suggesting the Fermi level of CdS in CT2.5 is lowered after its combination with Ti_3C_2. Thus, this result supports the electron transfer from CdS to Ti_3C_2 in CT2.5.

Supplementary Note 5. Discussion of the morphology and photocatalytic activity of Pt-CdS-1

The TEM image (Supplementary Fig. 27a) shows that the sizes of Pt NPs synthesized by the chemical-reduction method are in the range of 2-10 nm. Further observation on their HRTEM image (Supplementary Fig. 27b) indicates that Pt NPs exhibit the lattice spacings of 0.20 nm, corresponding to the (200) plane of face-centered cubic structured Pt (JCPDS No.04-0802). After loading 2.5 wt% Pt NPs on CdS SMSs, Pt-CdS-1 exhibits the similar morphology with that of CT2.5, except that Pt NPs instead of Ti_3C_2 NPs are loaded on the surface of CdS SMS (Supplementary Fig. 25a). The HRTEM image of Pt-CdS-1 (Supplementary Fig. 25b) shows the lattice spacings of 0.23 and 0.34 nm, in agreement with the (111) plane of face-centered cubic structured Pt and (002) plane of wurtzite-structured CdS, respectively. Hence, the contact between Pt and CdS is established in Pt-CdS-1, implying the possible charge transfer between Pt and CdS. Indeed, Pt-CdS-1 exhibits an obviously enhanced photocatalytic activity of 8234 μmol h^{-1} g^{-1} (Supplementary Fig. 26), compared to that of CT0 (105 μmol h^{-1} g^{-1}). This is attributed to the presence of ultra-small Pt NPs, which not only extract the photo-induced electrons from CdS, but also promote the H_2 evolution, as reported in many previous references.[13-15] Nevertheless, Pt-CdS-1 still exhibits lower photocatalytic activity than CT2.5 (14342 μmol h^{-1} g^{-1}). Given that these two samples show similar morphologies, the superior activity of CT2.5 should mainly arise from the stronger interaction between Ti_3C_2 NP and CdS SMS formed in the hydrothermal reaction,

compared to that between Pt NP and CdS SMS. This encouraging result demonstrates the great potential of Ti_3C_2 NP as a highly-active and economical substitute for Pt.

Supplementary Note 6. Analysis of the phase structures and optical properties of CN and CNT2.5

CT2.5, CN and CNT2.5 show almost the same XRD patterns (Supplementary Fig. 21b), which are consistent with the hexagonal wurtzite-structured CdS phase (JCPDS No. 77-2306). Moreover, the absorption edges of CN and CNT2.5 exhibit no apparent shift as compared with CT2.5 (Supplementary Fig. 21c), suggesting that Ni^{2+} is not doped into crystal structure of CdS in CN and CNT2.5. In fact, NiS is only loaded on the surface of CdS in these two samples.

Supplementary Note 7. Analysis of the phase structures and optical properties of ZCS and ZCS/Ti_3C_2

The XRD peaks of both ZCS and ZCS/Ti_3C_2 (Supplementary Fig. 23b) are clearly shifted to the left in comparison to standard cubic sphalerite-structured ZnS (JCPDS No. 05-0566), arising from the formation of $Zn_xCd_{1-x}S$ solid solution. After mixing with 1 wt.% Ti_3C_2 NPs, ZCS/Ti_3C_2 exhibits no obvious change in the XRD pattern compared to that of ZCS, due to the very low loading content (1 wt.%) of Ti_3C_2 NPs. Nevertheless, the UV-Vis diffuse reflectance spectrum of ZCS/Ti_3C_2 (Supplementary Fig. 23c) displays a significant increase in the 510-800 nm region compared with that of ZCS, due to the presence of black Ti_3C_2 NPs. Besides, the color of ZCS/Ti_3C_2 is also changed to olive from the yellow color of ZCS. The results clearly indicate the successful deposition of Ti_3C_2 NPs on ZCS in ZCS/Ti_3C_2.

Supplementary Note 8. Analysis of the phase structures and optical properties of ZnS and ZnS/Ti_3C_2

The cubic sphalerite-structured ZnS (JCPDS No. 05-0566) is observed for both ZnS and ZnS/Ti_3C_2 as shown in the XRD patterns (Supplementary Fig. 24b). Moreover, ZnS/Ti_3C_2 shows almost the same XRD pattern as that of ZnS, since the mechanical mixing of 1 wt.% Ti_3C_2 NPs with ZnS does not change its crystal structure. However, an obvious enhanced absorption in the 370-800 nm region is observed for the UV-Vis diffuse reflectance spectrum of ZnS/Ti_3C_2, in comparison to that of ZnS (Supplementary Fig. 24c). Also, the color of ZnS/Ti_3C_2 turned grey due to the loading of black-colored Ti_3C_2 NPs on the surface of white ZnS.

Supplementary Note 9. Discussion of the effect of F/O atomic ratio on photocatalytic activity

The surface F signal in CT0.05, CT0.1, CT5, CT7.5, CT2.5-E or CT2.5-5000 is negligible as examined by the XPS technique (Supplementary Fig. 28), indicating most of the F terminations on Ti_3C_2 in these samples were replaced by O or OH terminations during the hydrothermal reaction. Hence, the F/O atomic ratio on Ti_3C_2 should be zero in these samples. This result indicates that the H_2 evolution capacity and Fermi level position of Ti_3C_2 are optimized in these samples to boost their photocatalytic activities. Furthermore, this result excludes the influence of F/O ratio variation on the activity differences among these samples and CT2.5.

Supplementary Note 10. Analysis of the surface Pourbaix diagram of Ti_3C_2

At U_{SHE} = 0 V, the most stable state of Ti_3C_2 is terminated by 1/2 ML OH* and 1/2 ML O* (Supplementary Fig. 29a). As the pH decreases, the O* is combined with H^+ to form OH*. Under visible-light irradiation, the photo-induced electrons are supposed to transfer from CdS to Ti_3C_2 and further to the -OH terminations, where the adsorbed H^+ is reduced to evolve H_2 gas, again forming -O terminations. Based on the Pourbaix diagram (Supplementary Fig. 29b), the most stable termination of Ti_3C_2 is a mix of OH* and O*, which proves that our model for free energy calculation is reasonable.

Supplementary Note 11. Confirmation of the excellent conductivity of O-terminated Ti_3C_2 at different H coverages

As displayed in Supplementary Fig. 30a-d, continuous electronic states cross the Fermi level at all four H coverages, indicating that the conductivity of O-terminated Ti_3C_2 is excellent at all four H coverages.

Supplementary Methods

Experimental Section

Synthesis of Ti_3AlC_2. Elemental Ti (Alfa Aesar, Ward Hill, USA, 99 wt.% purity, particle size < 40 μm), Al (Alfa Aesar, Ward Hill, USA, 99 wt.% purity, particle size < 40 μm), and graphite (Alfa Aesar, Ward Hill, USA, 99 wt.% purity, particle size < 48 μm) powders were mixed with a molar ratio of 3 : 1.5 : 2. The mixture was ball-milled for 48 hours and cold pressed into cylindrical discs (15 mm in diameter and 10 mm in height) under 1 GPa pressure. The discs were put into a tube furnace under flowing Ar gas and heated to 1673 K for 2 hours at a ramp rate of 20 K min^{-1}. After cooling to room temperature, the discs were ball-milled for 2 hours to acquire fine powders for further investigation.

Synthesis of Ti_3C_2-E. Ti_3C_2-E was prepared by immersing Ti_3AlC_2 in 49% HF (Aladdin Reagent, China) at 333 k for 20 hours. All Al species in Ti_3AlC_2 were selectively removed by HF etching, while OH^-, O^- and/or F^- replaced Al as the terminal groups. The as-prepared sample was washed repeatedly with de-ionized water, centrifugally separated and dried in vacuum at 343 K for 8 hours.

Synthesis of Ti_3C_2 NPs. 100 mg of Ti_3C_2-E was added into 100 ml of de-ionized water and subjected to ultra-sonication for 5 hours, followed by centrifugation at 10000 RPM. After removal of the precipitates, a homogeneous dispersion of Ti_3C_2 NPs in the supernatant was obtained. The concentration of the obtained Ti_3C_2 NPs in aqueous solution was determined to be 0.15 mg ml^{-1}.

Synthesis of CdS/Ti_3C_2 composites. 0.368 g of $Cd(Ac)_2$ was firstly dissolved in de-ionized water after stirring. Then, a certain amount of Ti_3C_2 NPs in aqueous solution was added into the above solution under vigorous stirring. After 1 hour stirring, 0.42 g of thiourea was added into the suspension and stirred for 2 hours. Then the above suspension was transferred into a 50 ml autoclave and kept at 180 °C for 12 h. The obtained samples were washed twice with deionized water and ethanol, respectively, and dried at 333 K for 10 hours. The mass ratios of Ti_3C_2 to CdS were 0, 0.05, 0.1, 2.5, 5 and 7.5 wt%, and the resulting samples were labeled as CT0, CT0.05, CT0.1, CT2.5, CT5, and CT7.5, respectively.

Synthesis of Pt NPs. 7.5 ml of 0.1 M CTAB aqueous solution was dissolved in warm water, followed by adding 2.5 ml of 1 mM H_2PtCl_6 aqueous solution and 0.6 ml of 10 mM $NaBH_4$ aqueous

solution under vigorous stirring. Then the above solution was transfered to oil bath at 120 °C. After stirring for 15 min, the synthesized products were cooled down to room temperature, centrifuged to remove the redundant CTAB in the aqueous solution and stored in fridge. The concentration of the Pt NPs was determined by thermogravimetric analysis.

Synthesis of CNT2.5. 0.368 g of $Cd(Ac)_2$ and 0.003 g of $Ni(Ac)_2$ were firstly dissolved in de-ionized water after stirring. Subsequently, a certain amount of Ti_3C_2 NPs in aqueous solution was added into the above solution under vigorous stirring. After 1 hour of stirring, 0.42 g of thiourea was added into the suspension and stirred for 2 hours. Then the above suspension was transferred into a 50 ml autoclave and kept at 180 °C for 12 h. The as-prepared sample was washed twice by deionized water and ethanol, respectively, and then dried at 333 K for 10 hours.

Synthesis of ZnS. 0.633 g of $Zn(Ac)_2 \cdot 2H_2O$ was added into 31 ml of deionized water and 5 ml of 0.9 M Na_2S aqueous solution was then added dropwise into this suspension followed by stirring for 6 h at room temperature. The resulting suspension was then transferred into a 50 ml autoclave and maintained at 180 °C for 12 h. The acquired sample was washed twice with deionized water and ethanol, respectively, and then dried at 333 K for 10 hours.

Computation Section

Active sites and H adsorption properties. The differential Gibbs free energy of the adsorption of atomic H ($\Delta G^0_{H^*}$) is obtained by Eq (1):

$$\Delta G^0_{H^*} = \Delta E_H + \Delta E_{ZPE} - T\Delta S_H \quad (1)$$

where ΔE_{ZPE} and ΔS_H are the changes in the zero point energy and entropy between H adsorption and H_2 in the gas phase, respectively. The contributions from the catalysts to both ΔE_{ZPE} and ΔS_H are considered negligible. Therefore, ΔE_{ZPE} is obtained by Eq (2)[16]:

$$\Delta E_{ZPE} = E^{nH}_{ZPE} - E^{(n-1)H}_{ZPE} - {}^1\!/_2\, E^{H_2}_{ZPE} \quad (2)$$

where E^{nH}_{ZPE} is the zero point energy of n-adsorbed H atoms on the catalyst without the contribution of catalyst and $E^{H_2}_{ZPE}$ is the zero point energy of H_2 in gas phase. The vibration frequency of H adsorption on Ti_3C_2, O-terminated Ti_3C_2 or F-terminated Ti_3C_2 at 1/8 coverage is 3705.0 cm^{-1}, and

not sensitive to the coverages. The calculated frequency of H_2 gas is 4289.4 cm^{-1}. ΔS_H is obtained by Eq (3):

$$\Delta S_H \cong -{}^{1}\!/_{2}\, S^0_{H_2} \tag{3}$$

where $S^0_{H_2}$ is the entropy of H_2 gas at standard conditions.

Therefore, Eq (1) can be rewritten as Eq (4):

$$\Delta G^0_{H^*} = \Delta E_H + 0.30\ \text{eV} \tag{4}$$

where ΔE_H is the differential H adsorption energy, which is defined by Eq (5):

$$\Delta E_H = E_{nH*} - E_{(n-1)H*} - {}^{1}\!/_{2}\, E_{H_2} \tag{5}$$

where * denotes the catalyst. E_{nH*}, $E_{(n-1)H*}$ and E_{H_2} represent the total energies of catalyst with n adsorbed H atoms, total energies of catalyst plus (n-1) adsorbed H atoms and H_2 gas, respectively.

Fermi level positions. The LVTOT in VASP is firstly applied to obtain the Fermi Level *vs.* vacuum level and the Fermi level *vs.* SHE is then calculated using the following Eq (6):

$$E_F(vs.\ \text{SHE}) = -4.44\ \text{V} - E_F(vs.\ \text{vaccum level}) \tag{6}$$

Surface Pourbaix Diagrams. The surface Pourbaix diagrams of Ti_3C_2 were constructed by plotting the most stable state of the surface under relevant U_{SHE} and pH. In our model, we assumed that the oxidation of water to OH* and O* on Ti_3C_2 through the following steps as suggested by reference[17]:

$$H_2O + * \rightarrow OH^* + H^+ + e^- \tag{7}$$

$$OH^* \rightarrow O^* + H^+ + e^- \tag{8}$$

Under standard conditions, the free energy of $H^+ + e^-$ is equal to ½ H_2. Therefore, Eq (7) and Eq (8) can be rewritten into Eq (9) and Eq (10):

$$H_2O + * \rightarrow OH^* + \frac{1}{2} H_2 \tag{9}$$

$$OH^* \rightarrow O^* + \frac{1}{2} H_2 \tag{10}$$

The Gibbs free-energies of Eq (9) ($\Delta G^0_{OH^*}$) and Eq (10) ($\Delta G^0_{O^*}$) are obtained by Eq (11):

$$\Delta G^0 = \Delta E + \Delta E_{ZPE} - T\Delta S \tag{11}$$

Where, ΔE is the energy difference of Eq (9) or Eq (10). The values for ΔE_{ZPE}-$T\Delta S$ are calculated from the value table I of reference[18].

Both Eq (7) and Eq (8) are dependent on the pH and potential U through the chemical potential of H^+ and e^-, respectively, while Eq (9) and Eq (10) are not. To include the effects of pH and potential U, the Eq(11) are rewritten into Eq (12) and Eq (13):

$$\Delta G_{OH^*} = \Delta G^0_{OH^*} - eU_{SHE} - k_b T \ln 10 \times pH \tag{12}$$

$$\Delta G_{O^*} = \Delta G^0_{O^*} - eU_{SHE} - k_b T \ln 10 \times pH \tag{13}$$

Based on Eq (12) and Eq (13), we can calculate the free energy of Ti_3C_2 with different coverages of OH* and O* terminations under different conditions. The free energy of Ti_3C_2 with mixture terminations ($Ti_3C_2(OH)_x(O)_y$, $x + y \leq 2$) are obtained by Eq (14):

$$\Delta G_{mix} = \Delta G^0_{mix} - (x + 2y)U_{SHE} - (x + 2y)k_b T \ln 10 \times pH \tag{14}$$

Therefore, we can obtain the free energy of Ti_3C_2 under different OH* and O* coverages. The most stable state of the surface under relevant conditions is used to construct the surface Pourbaix diagrams.

Supplementary References

1. Wang, H. *et al.* Nickel nanoparticles modified CdS - A potential photocatalyst for hydrogen production through water splitting under visible light irradiation. *Int. J. Hydrogen Energy* **40,** 340-345 (2015).
2. Chen, X. *et al.* In situ photodeposition of NiO_x on CdS for hydrogen production under visible light: Enhanced activity by controlling solution environment. *Appl. Catal. B-Environ.* **152-153,** 68-72 (2014).
3. Ran, J., Yu, J. & Jaroniec, M. $Ni(OH)_2$ modified CdS nanorods for highly efficient visible-light-driven photocatalytic H_2 generation. *Green Chem.* **13**, 2708-2713 (2011).
4. Zhang, J., Qiao, S. Z., Qi, L. & Yu, J. Fabrication of NiS modified CdS nanorod p-n junction photocatalysts with enhanced visible-light photocatalytic H_2-production activity. *Phys. Chem. Chem. Phys.* **15,** 12088-12094 (2013).
5. Cao, S. *et al.* Spectacular photocatalytic hydrogen evolution using metal-phosphide/CdS hybrid catalysts under sunlight irradiation. *Chem. Commun.* **51,** 8708-8711 (2015).
6. Yuan, J. *et al.* Amorphous Co_3O_4 modified CdS nanorods with enhanced visible-light photocatalytic H_2-production activity. *Dalton Trans.* **44,** 1680-1689 (2015).
7. Peng, T. *et al.* Enhanced photocatalytic hydrogen production over graphene oxide-cadmium sulfide nanocomposite under visible light irradiation. *J. Phys. Chem. C* **116,** 22720-22726 (2012).
8. Ye, A. *et al.* CdS-graphene and CdS-CNT nanocomposites as visible-light photocatalysts for hydrogen evolution and organic dye degradation. *Catal. Sci. Technol.* **2,** 969-978 (2012).
9. Lang, D., Shen, T. & Xiang, Q. Roles of MoS_2 and graphene as cocatalysts in the enhanced visible-light photocatalytic H_2 production activity of multiarmed CdS nanorods. *ChemCatChem* **7,** 943-951 (2015).
10. Chen, J. *et al.* One-pot synthesis of CdS nanocrystals hybridized with single-layer transition-metal dichalcogenide nanosheets for efficient photocatalytic hydrogen evolution. *Angew. Chem. Int. Ed.* **54,** 1210-1214 (2015).
11. Naguib, M. *et al.* Two-dimensional nanocrystals produced by exfoliation of Ti_3AlC_2. *Adv. Mater.* **23,** 4248-4253 (2011).
12. Yan, P. *et al.* Enhanced supercapacitive performance of delaminated two-dimensional titanium

carbide/carbon nanotube composites in alkaline electrolyte. *J. Power Sources* **284,** 38-43 (2015).

13. Bao, N., Shen, L., Takata, T. & Domen, K. Self-templated synthesis of nanoporous CdS nanostructures for highly efficient photocatalytic hydrogen production under visible light. *Chem. Mater.* **20,** 110-117 (2008).
14. Darwent, J. R. & Porter, G. Photochemical hydrogen production using cadmium sulphide suspensions in aerated water. *J. Chem. Soc., Chem. Commun.* 145-146 (1981).
15. Yan, H. *et al.* Visible-light-driven hydrogen production with extremely high quantum efficiency on Pt-PdS/CdS photocatalyst. *J. Catal.* **266**, 165-168 (2009).
16. Tsai, C., Abild-Pedersen, F. & Nørskov, J. K. Tuning the MoS_2 edge-site activity for hydrogen evolution via support interactions. *Nano Lett.* **14**, 1381-1387 (2014).
17. Hansen, H. A., Rossmeisl, J. & Norskov, J. K., Surface Pourbaix diagrams and oxygen reduction activity of Pt, Ag and Ni(111) surfaces studied by DFT. *Phys. Chem. Chem. Phys.* **10,** 3722-3730 (2008).
18. Valdés, Á. *et al.* Oxidation and photo-oxidation of water on TiO_2 surface. *J. Phys. Chem. C* **112,** 9872-9879 (2008).
